# The causal relationship between sarcopenia‐related traits and ischemic stroke: Insights from univariable and multivariable Mendelian randomization analyses

**DOI:** 10.1111/cns.14759

**Published:** 2024-05-16

**Authors:** Jiahao Song, Da Zhou, Jingrun Li, Mengqi Wang, Lina Jia, Duo Lan, Haiqing Song, Xunming Ji, Ran Meng

**Affiliations:** ^1^ Department of Neurology, Xuanwu Hospital Capital Medical University Beijing China; ^2^ Advanced Center of Stroke Beijing Institute for Brain Disorders Beijing China; ^3^ National Center for Neurological Disorders, Xuanwu Hospital Capital Medical University Beijing China

**Keywords:** appendicular lean mass, ischemic stroke, mediators, Mendelian randomization, sarcopenia

## Abstract

**Aims:**

The causal relationship between sarcopenia‐related traits and ischemic stroke (IS) remains poorly understood. This study aimed to explore the causal impact of sarcopenia‐related traits on IS and to identify key mediators of this association.

**Methods:**

We conducted univariable, multivariable two‐sample, and two‐step Mendelian randomization (MR) analyses using genome‐wide association study (GWAS) data. This included data for appendicular lean mass (ALM), hand grip strength (HGS), and usual walking pace (UWP) from the UK Biobank, and IS data from the MEGASTROKE consortium. Additionally, 21 candidate mediators were analyzed based on their respective GWAS data sets.

**Results:**

Each 1‐SD increase in genetically proxied ALM was associated with a 7.5% reduction in the risk of IS (95% CI: 0.879–0.974), and this correlation remained after controlling for levels of physical activity and adiposity‐related indices. Two‐step MR identified that six mediators partially mediated the protective effect of higher ALM on IS, with the most significant being coronary heart disease (CHD, mediating proportion: 39.94%), followed by systolic blood pressure (36.51%), hypertension (23.87%), diastolic blood pressure (15.39%), type‐2 diabetes mellitus (T2DM, 12.71%), and low‐density lipoprotein cholesterol (7.97%).

**Conclusion:**

Our study revealed a causal protective effect of higher ALM on IS, independent of physical activity and adiposity‐related indices. Moreover, we found that higher ALM could reduce susceptibility to IS partially by lowering the risk of vascular risk factors, including CHD, hypertension, T2DM, and hyperlipidemia. In brief, we elucidated another modifiable factor for IS and implied that maintaining sufficient muscle mass may reduce the risk of such disease.

## Graphical abstract

This study demonstrated that higher appendicular lean mass could reduce susceptibility to ischemic stroke, partially by lowering the risk of cardiovascular factors, such as coronary heart disease and hypertension. Preservation of muscle mass may potentially be a therapeutic intervention in stroke patients.

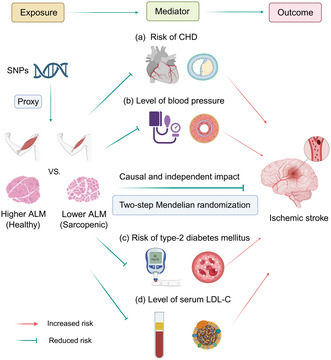

## 1 INTRODUCTION

Stroke, the most prevalent form of cerebrovascular disease, poses a major threat to global health. Epidemiologically, it ranks as the second leading cause of death worldwide, accounting for approximately 11.6% of all deaths, and as the third leading cause of death and disability combined, responsible for about 5.7% of total disability‐adjusted life years (DALYs).^1^, ^2^ Stroke is primarily classified into ischemic and hemorrhagic types, with ischemic stroke (IS) constituting 62.4%–87% of cases.^3^, ^4^, ^5^ The lifetime risk of experiencing a stroke and IS in adults over the age of 25 is estimated at 24.9% and 18.3%, respectively.^6^ With the aging population, IS cases are projected to increase to 9.62 million by 2030.^1^ Fortunately, research over the past four decades has shown that up to 90% of IS cases are preventable, with well‐known modifiable risk factors such as hypertension, type 2 diabetes (T2DM), and dyslipidemia identified.^7^, ^8^ However, these factors do not fully explain the diverse nature of IS,^8^ underscoring the need to identify additional risk factors for more effective prediction and prevention.

Sarcopenia, an age‐related decline in muscle mass and function, has emerged as a significant but underrecognized factor potentially impacting IS.^9^ This condition, characterized by progressive muscle deterioration, is associated with several adverse outcomes, including fragility, fractures, falls, and even mortality,^10^ affecting 10%–16% of the elderly population globally.^11^ To better identify sarcopenia and quantify its severity, the European Working Group on Sarcopenia in Older People (EWGSOP) has proposed an intact assessment system based on muscle strength, muscle mass, and physical performance. Although some clinical studies suggest a link between sarcopenia and IS,^12^, ^13^, ^14^, ^15^ these often involve individuals with metabolic dysfunctions, thereby introducing selection bias. The challenge of discerning this relationship is further compounded by the potential for residual confounding from factors like body mass index (BMI), physical activity (PA), and adiposity‐related indices, as well as the possibility of reverse causation linked to co‐existing conditions such as cardiovascular disease (CVD), osteoporosis, or cancers.

To address these complexities, Mendelian randomization (MR) studies, which utilize genetic variations, namely single nucleotide polymorphisms (SNPs) to investigate the effects of exposures on outcomes, offer a robust alternative. Leveraging the increasing availability of genome‐wide association study (GWAS) data, MR provides a method to explore causal relationships while controlling for confounding and reverse causation. This approach can yield insights comparable in quality to those derived from randomized controlled trials. Given these advantages, we employed a comprehensive MR analysis to unearth the potential causal relationship between sarcopenia‐related phenotypes and the risk of IS.

## 2 MATERIALS AND METHODS

### 2.1 Study design

**FIGURE 1:**
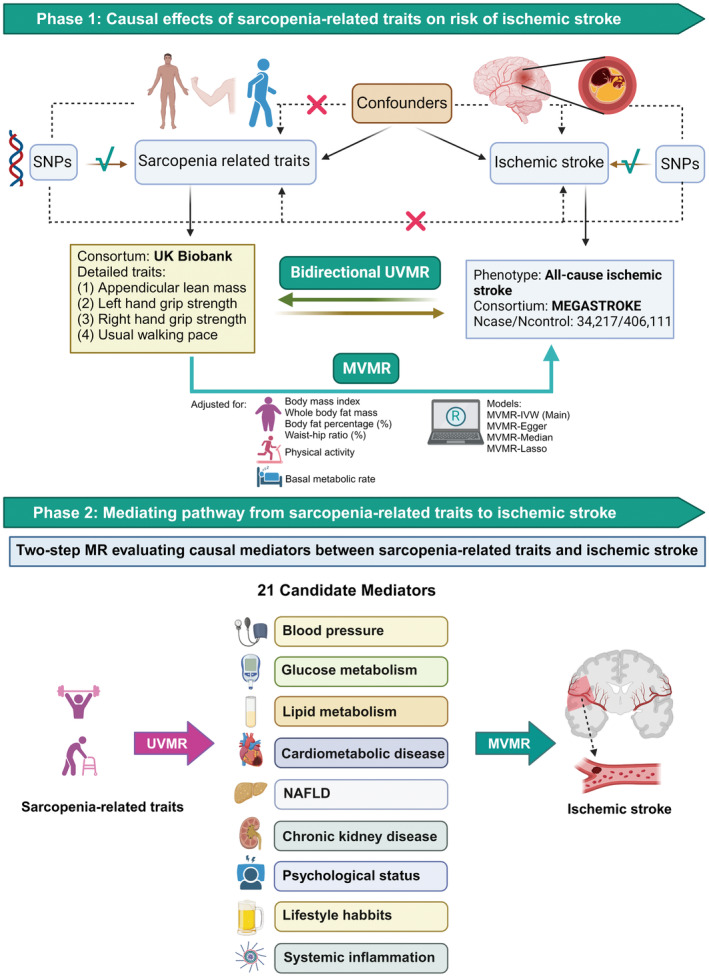
Overview of the MR study. This MR study comprised two analytical phases. In phase 1, UVMR estimated the causal associations between sarcopenia‐related traits and IS, suggesting that higher ALM may causally reduce the risk of IS. In phase 2, two‐step MR was performed to screen for 21 candidate mediators potentially underlying the pathways between ALM and IS, and the mediation effects and proportions for qualified mediators were quantified. ALM, appendicular lean mass; MR, Mendelian randomization; MVMR, multivariable Mendelian randomization; MV‐IVW, multivariable inverse variance weighted model; NAFLD, nonalcoholic fatty liver disease; SNPs, single nucleotide polymorphisms; UVMR, univariable Mendelian randomization (figure was created with BioRender.com).

This MR study comprised two analysis stages (Figure 1). In the first stage, we conducted a two‐sample bidirectional MR to assess whether sarcopenia‐related traits had a causal impact on IS or vice versa. Subsequently, multivariable MR (MVMR) was performed to evaluate the independent effects of appendicular lean mass (ALM) on IS risk, after adjusting for some body‐fat indices and PA. In the second stage, we selected 21 candidate mediators in the pathway from ALM to IS. A two‐step MR analysis was then utilized to estimate the mediating effects of each screened mediator on the causal relationship between ALM and IS risk. Our analytical processes and reporting procedures strictly adhered to the three core assumptions of MR and STROBE‐MR guidelines, respectively.^16^, ^17^

### 2.2 Selection of genetic variants

The selection of instrumental variables (IVs) was rigorously based on the core assumptions of MR. To satisfy the relevance assumption, we first selected SNPs strongly associated with each exposure and mediator from their corresponding GWASs, using a significance threshold of *p* < 5E‐08. For the reverse causality analysis between sarcopenia and IS, a more lenient threshold of *p* < 5E‐07 was applied, due to the scarcity of SNPs meeting the stricter criterion. Subsequently, to obtain independent IVs, a clumping procedure was executed with a cut‐off point of *r* ^2^ < 0.001 and clumping window size = 10,000 kb. Then *R* ^2^ and *F*‐statistics of each SNP were calculated to preclude the possibility of weak instrument bias. SNPs with *F*‐statistics < 10 were considered as weak IVs and dismissed from the dataset. To adhere to the exclusivity assumption, SNPs directly associated with IS (*p* < 5E‐08) were discarded. Sensitivity analyses were conducted to test for and exclude pleiotropic effects (as detailed in the Statistical analyses section). For the independence assumption, MVMR analysis was employed to ascertain the independent effects of each variable, adjusting for potential confounders. Moreover, proxy SNPs were utilized in our study if certain IVs were not extracted from the outcome GWAS. Finally, a harmonization procedure was performed to align exposure and outcome SNPs, while SNPs featuring incompatible alleles and palindromic SNPs with intermediate EAF were removed.

### 2.3 Data sources

#### 2.3.1 Data sources for exposures and outcomes

Detailed information about the data sources for each phenotype in our analysis is presented in Table 1. Sarcopenia assessment was based on three primary criteria: muscle mass, muscular strength, and physical performance. We chose ALM as an indicator of muscle mass, hand grip strength (HGS) for measuring muscular strength, and usual walking pace (UWP) to represent physical performance. The IVs for ALM (*n* = 450,243), left HGS (*n* = 461,026), right HGS (*n* = 461,089), and UWP (*n* = 459,915) were all extracted from GWAS data sets derived from the UK Biobank, which is a large prospective cohort consisted of over 500,000 European individuals.^18^ Outcome data for IS (N_cases = 34,217, N_controls = 406,111), adhering to WHO clinical and imaging criteria, were sourced from the MEGASTROKE consortium.^19^ There was no sample overlap between the data sets used for exposures and outcomes, ensuring the independence of our analysis.

**TABLE 1:** GWAS data sources in this MR study.

Phenotype	PMID	Sample size or case/control	Ancestry	Unit	Consortium
Exposure					
ALM	33,097,823	450,243	European	1‐SD	UK Biobank
Left HGS	29,846,171	461,026	European	1‐SD	UK Biobank
Right HGS	29,846,171	461,089	European	1‐SD	UK Biobank
UWP	29,846,171	459,915	European	1‐SD	UK Biobank
Confounders					
BMI	30,124,842	681,275	European	1‐SD	GIANT
WBFM	29,846,171	454,137	European	1‐SD	UK Biobank
BF%	34,017,140	401,772	European	1‐SD	UK Biobank
WHR	29,892,013	502,773	European	1‐SD	UK Biobank
PA	29,899,525	91,084	European	1‐SD	UK Biobank
BMR	29,846,171	454,874	European	1‐SD	UK Biobank
Mediators					
Hypertension	29,846,171	42,857/162,837	European	Event	FinnGen
SBP	30,224,653	757,601	European	1 mmHg	ICBP
DBP	30,224,653	757,601	European	1 mmHg	ICBP
T2DM	34,594,039	38,841/451,248	European	Event	Saori Sakaue's
Fasting insulin	34,059,833	151,013	European	Logtransformed pmol/L	MAGIC
HbA1c	34,059,833	146,806	European	%	MAGIC
Fasting glucose	34,059,833	200,622	European	1 mmol/L	MAGIC
2‐hour glucose	34,059,833	63,396	European	Logtransformed mmol/L	MAGIC
TC	24,097,068	188,577	Mixed (96% European)	1‐SD	GLGC
LDL‐C	24,097,068	188,577	Mixed (96% European)	1‐SD	GLGC
HDL‐C	24,097,068	188,577	Mixed (96% European)	1‐SD	GLGC
TG	24,097,068	188,577	Mixed (96% European)	1‐SD	GLGC
AF	28,416,818	15,979/102,776	European	Event	AFGen
CHD	26,343,387	60,801/123,504	Mixed (77% European)	Event	CARDIoGRAMplusC4D
NAFLD	29,846,171	894/217,898	European	Event	FinnGen
Depression	30,718,901	170,756/329,443	European	Event	PGC
Anxiety	29,846,171	20,992/197,800	European	Event	FinnGen
Chronic kidney disease	29,846,171	3902/212,841	European	Event	FinnGen
Cigarettes per day	30,643,251	337,334	European	1‐SD	GSCAN
Alcoholic drinks per week	30,643,251	337,334	European	1‐SD	GSCAN
CRP	35,459,240	575,531	European	1‐SD	Saredo Said's
Outcome					
Ischemic stroke	29,531,354	34,217/406,111	European	Event	MEGASTROKE

Abbreviations: AF, atrial fibrillation; AFGen, atrial fibrillation genetics; ALM, appendicular lean mass; BF%, body fat percentage; BMI, body mass index; BMR, basal metabolic rate; CARDIoGRAMplusC4D, Coronary ARtery DIsease Genome‐wide Replication and Meta‐analysis plus The Coronary Artery Disease Genetics; CHD, coronary heart disease; CRP, C‐reactive protein; DBP, diastolic blood pressure; GLGC, Global Lipids Genetics Consortium; GSCAN, GWAS and Sequencing Consortium of Alcohol and Nicotine use; HGS, hand grip strength; HDL‐C, High‐density lipoprotein cholesterol; ICBP, International Consortium of Blood Pressure; LDL‐C, low‐density lipoprotein cholesterol; MAGIC, meta‐analyses of glucose‐ and insulin‐related traits; MEGASTROKE, multi‐ancestry Genome‐Wide Association Study of Stroke; NAFLD, nonalcoholic fatty liver disease; PA, physical activity; PGC, Psychiatric Genomics Consortium; PMID, the PubMed ID; SBP, systolic blood pressure; SD, standard deviation; T2DM, type‐2 diabetes mellitus; TC, total cholesterol; TG, Triglyceride; UWP, usual walking pace; WBFM, whole body fat mass; WHR, waist–hip ratio.

#### 2.3.2 Data sources for confounders

To mitigate the limitations inherent in observational studies, we considered a range of body composition parameters as potential confounding factors. We utilized data from the GIANT consortium for BMI (*n* = 681,275), and additional metrics from the UK Biobank, including whole‐body fat mass (WBFM, *n* = 454,137), body fat percentage (BF%, *n* = 401,772), waist‐to‐hip ratio (WHR, *n* = 502,773), level of PA (*n* = 91,084), and basal metabolic rate (BMR, *n* = 454,874). These variables were selected for their potential to influence both sarcopenia and IS risk, thereby controlling for confounding in our analysis.

#### 2.3.3 Data sources for potential mediators

Given the deleterious effects of low body lean mass and sarcopenia on metabolic homeostasis—impacting blood pressure regulation, glucose and lipid metabolism, chronic inflammation, psychological health, and various metabolic disorders—we identified these areas as candidate mediators in the causal pathway from ALM to IS. More specifically, the potential mediators include (1) three traits of blood pressure: hypertension (*n* = 205,694) from the FinnGen Biobank, and both systolic blood pressure (SBP) and diastolic blood pressure (DBP) from the ICBP consortium (*n* = 757,601)^20^; (2) five insulin and glycemic traits: T2DM (*n* = 490,089),^21^ fasting insulin (FI, *n* = 151,013), HbA1c (*n* = 146,806), fasting glucose (*n* = 200,622), and two‐hour postprandial glucose (THG, *n* = 63,396) from the MAGIC consortium^22^; (3) four lipid traits: total cholesterol (TC), low‐density lipoprotein cholesterol (LDL‐C), high‐density lipoprotein cholesterol, and triglyceride (TG), all derived from the GLGC consortium (*n* = 188,577)^23^; (4) two cardiometabolic diseases: coronary heart disease (CHD, *n* = 184,305) from the CARDIoGRAMplusC4D,^24^ and atrial fibrillation (AF, *n* = 118,755) from the AFGen consortium^25^; (5) non‐alcoholic fatty liver disease (NAFLD, *n* = 218,792) from the FinnGen Biobank; (6) chronic kidney disease (*n* = 216,743) from the FinnGen Biobank; (7) two lifestyle habits: smoking (*n* = 337,336) and alcoholic drinking (*n* = 335,394) from the GSCAN consortium^26^; (8) two psychological states: depression (*n* = 500,199) and anxiety (*n* = 218,792) from the PGC^27^ and FinnGen Biobanks, respectively; and (9) one inflammatory marker: C‐reactive protein (CRP, *n* = 575,531) from Said S's GWAS.^28^

### 2.4 Statistical analyses

#### 2.4.1 Univariable and multivariable MR analyses

For univariable MR (UVMR) analysis, the random‐effect inverse‐variance weighted (IVW) method was regarded as the primary model to ascertain the causal relationship between four sarcopenia‐related traits and IS. The IVW method synthesized Wald ratios from each SNP into a composite estimated effect via meta‐analysis. In addition to IVW, four alternative approaches—maximum likelihood, weighted median, MR‐Egger, and MR‐PRESSO—were employed to validate the robustness of the results. Causal inference was considered statistically significant if both the following criteria were met: (1) p_ivw_< p_threshold_ and (2) consistent direction of effect estimates across all methods.

To control for type I errors stemming from multiple testing, we applied the Bonferroni correction, setting a significance threshold at 0.0125 (0.05/4). Cochran's Q test assessed heterogeneity, with a Q *p*‐value < 0.05 indicating its presence. To evaluate horizontal pleiotropy, the MR‐Egger intercept and intercept *p*‐value were calculated. An intercept *p*‐value of <0.05 would cast doubt on the causal inference drawn from our MR analysis. Additionally, the Steiger directional test was used to measure any potential reverse causation bias in the newly established causal relationship.

For MVMR analysis, multivariable inverse variance weighted model (MV‐IVW) was utilized as the primary analytical model, with MVMR‐Egger, MVMR‐median, and MVMR‐Lasso as supplementary approaches. The MVMR‐Egger intercept and its *p*‐value were computed as key parameters to evaluate horizontal pleiotropy.

#### 2.4.2 Effect of sarcopenia‐related traits on IS

UVMR was conducted to estimate the causal impacts of ALM, left HGS, right HGS, and UWP on IS. Due to detected heterogeneity, a random‐effect IVW model was adopted. Radial MR was then applied to identify and exclude outliers, followed by a reanalysis to validate the initial results. Additionally, a reverse two‐sample MR analysis was executed to explore if IS could causally increase the burden of sarcopenia.

As parameters such as BMI, WBFM, BF%, WHR, level of PA, and BMR are closely correlated with both sarcopenia‐related traits and the risk of IS, they were considered as potential confounders in this study. MVMR was then performed to ascertain the independent effect of muscular traits on IS with adjustment for these adipose‐related indices.

#### 2.4.3 Selection of mediators and two‐step MR

We screened potential mediators in the causal pathway linking sarcopenia and IS based on the following criteria: (1) a causal correlation between ALM and each mediator; (2) a causal association between each mediator and IS, both with and without adjustment for ALM; (3) absence of reverse causation between the mediators and ALM; and (4) consistency in the direction of the mediating effects of each mediator with the overall effect of ALM on IS. A total of six mediators meeting these criteria were included in subsequent mediation analyses. The mediation analysis was conducted in two steps using the two‐step MR approach. Initially, UVMR estimated the causal impacts (β1) of genetically proxied ALM on the mediators. Subsequently, MVMR assessed the independent effects (β2) of mediators on IS with adjustment for ALM. The mediating effect (β1 × β2) of each mediator was generated from the “product‐of‐coefficients” approach, and the proportion of mediation was calculated by dividing the mediating effect (β1 × β2) by the total effect (β0). Standard errors for both the mediating effects and proportions were determined using the delta method. All analyses were performed using the TwoSampleMR package (version 0.5.7), MendelianRandomization package (version 0.9.0), and RadialMR (version 1.1) in R software (version 4.3.1).

## 3 RESULTS

### 3.1 Univariable bidirectional MR analyses for the causal association between four muscular traits and IS

**FIGURE 2:**
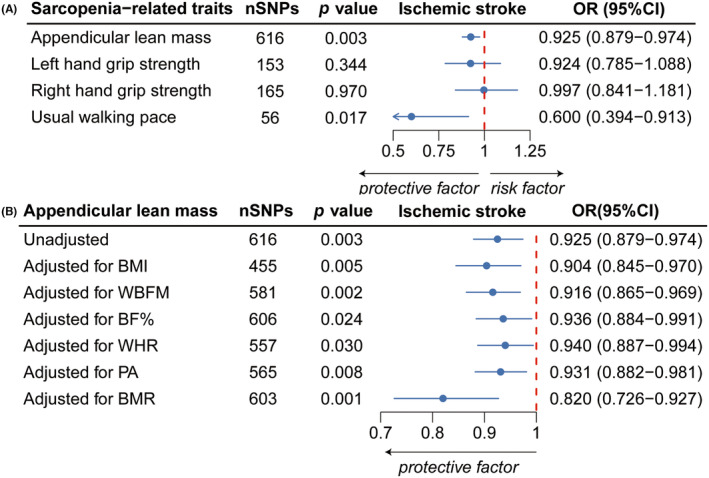
Forest plots of the association between sarcopenia‐related traits and IS. (A) Forest plot presenting the causal estimates between sarcopenia‐related traits and IS as determined by UVMR. (B) Forest plot showing the causal estimates between ALM and IS, before and after adjustments for BMI, WBFM, BF%, WHR, levels of PA, and BMR using MVMR. ALM, appendicular lean mass; BMI, body mass index; BF%, body fat percentage; BMR, basal metabolic rate; IS, ischemic stroke; MVMR, multivariable Mendelian randomization; OR, odds ratio; PA, physical activity; SNPs, single nucleotide polymorphisms; UVMR, univariable Mendelian randomization; WBFM, whole‐body fat mass; WHR, waist‐to‐hip ratio.

Univariable forward MR analyses showed that each 1‐SD increase in genetically proxied ALM was associated with a 7.5% reduction in the risk of IS (OR = 0.925, 95% CI: 0.879–0.974, *p* = 0.003) (Figure 2A). However, no causal associations were observed between left HGS (OR = 0.924, 95% CI: 0.785–1.088, *p* = 0.344), right HGS (OR = 0.997, 95% CI: 0.841–1.181, *p* = 0.970), or UWP (OR = 0.600, 95% CI: 0.394–0.913, *p* = 0.017) and IS (Figure 2A). MR‐Egger intercept statistics revealed no evidence for horizontal pleiotropy (all p_intercept_ ≥ 0.435), and Steiger tests confirmed no reverse causation bias (all Steiger *p*‐value ≤ 1.25E‐152) (Tables S2 and S3). Despite potential heterogeneity detected by Cochran's Q test (Table S2), subsequent analyses using Radial MR to remove 54 outliers reaffirmed the significant causal impact of ALM on IS (OR = 0.935, 95% CI: 0.893–0.979, *p* = 0.005) (Table S4). Conversely, reverse MR analyses did not demonstrate any significant causal effects of IS on the four sarcopenia‐related traits (all p_ivw_ ≥ 0.073) (Table S6).

### 3.2 MVMR analyses for the causal and independent effect of muscular traits on IS

Our MVMR analysis established a causal relationship between ALM and IS, which persisted even after adjusting for BMI, WBFM, BF%, WHR, level of PA, and BMR, with OR (95% CI) ranging from 0.820 (95% CI: 0.726–0.927) to 0.940 (95% CI: 0.887–0.994) (Figure 2B). Supplementary methods including MVMR‐Egger, MVMR‐median, and MVMR‐Lasso further validated the results derived from the MV‐IVW model (Table S8). MVMR‐Egger intercept tests showed no horizontal pleiotropy, enhancing the reliability of our estimates.

### 3.3 UVMR analyses for the causal impact of sarcopenia‐related traits on potential mediators

**FIGURE 3:**
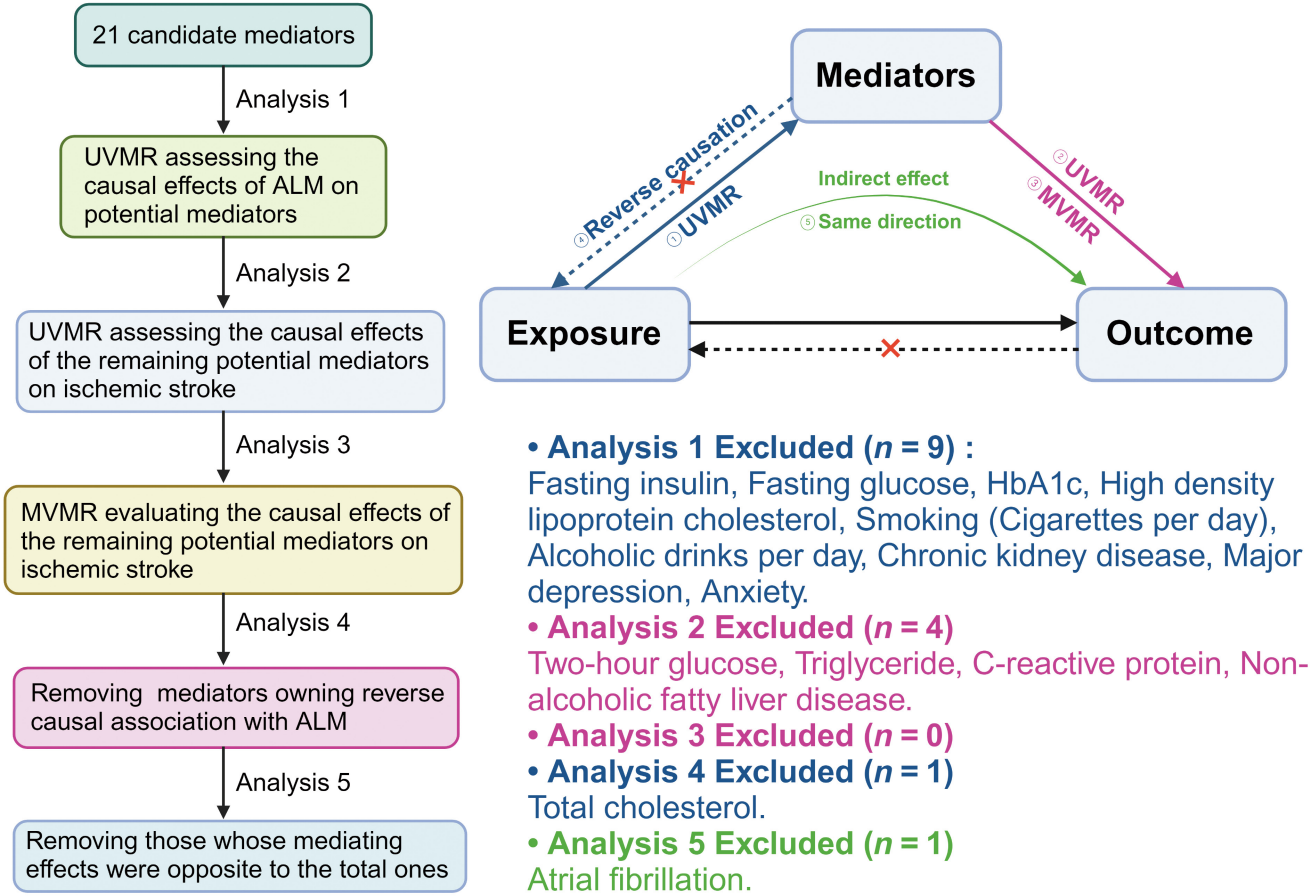
Screening process for mediators in the causal correlation of ALM with IS. Five analytical criteria were performed to select mediators in the causal pathway from higher ALM to IS: (i) causal association of ALM with mediators; (ii) mediator's causal effect on the outcome via UVMR; (iii) direct causal effect of mediators on IS independent of ALM from MVMR; (iv) absence of reverse causation with ALM; and (v) alignment of total and mediating effects of ALM on IS. Eventually, six mediators meeting the above criteria were included in further analyses to quantify their individual mediating contributions. ALM, appendicular lean mass; IS, ischemic stroke; MVMR, multivariable Mendelian randomization (figure was created with BioRender.com).

**FIGURE 4:**
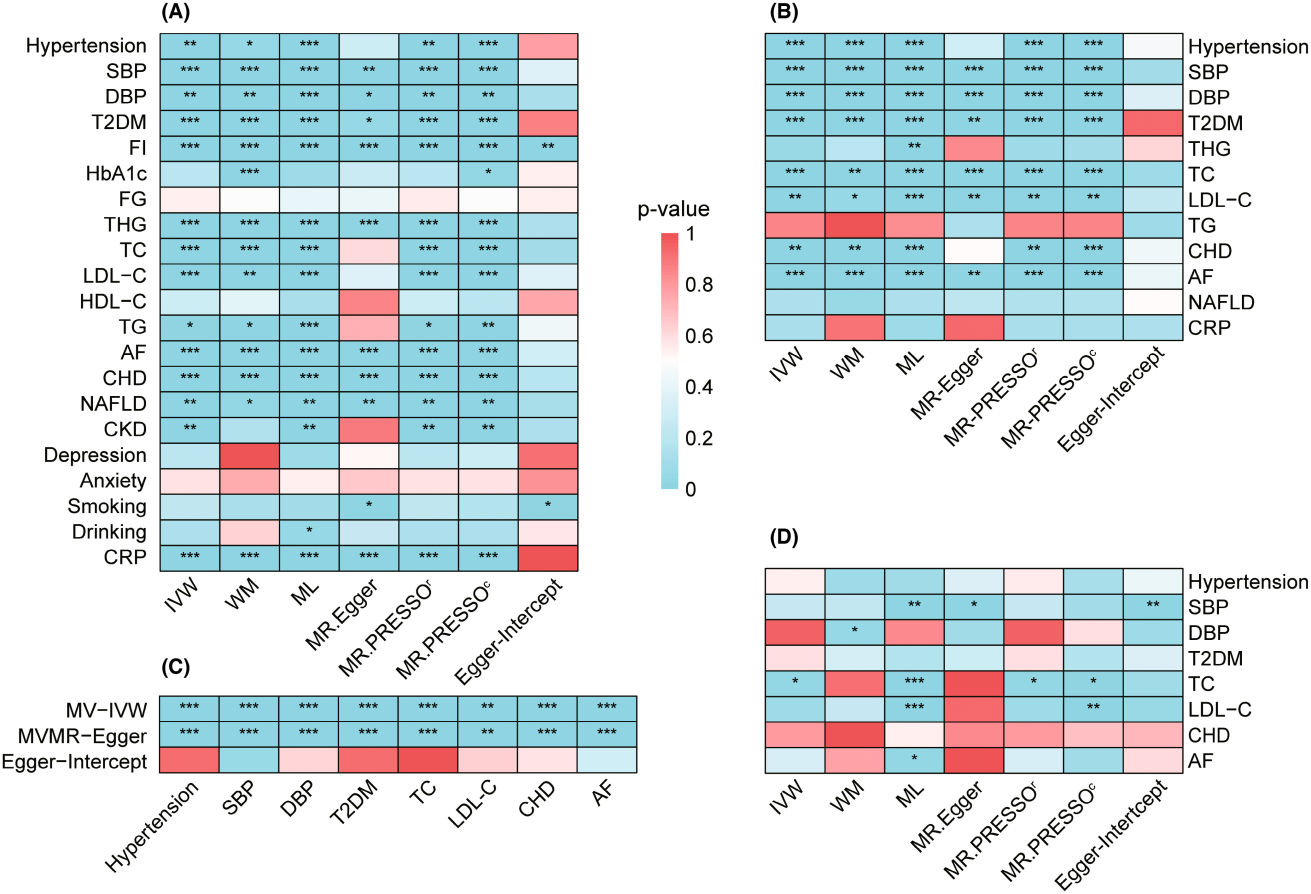
Overall MR estimates for the causal impacts. (A) Causal effects of ALM on potential mediators determined by UVMR; (B) causal effects of remaining candidate mediators on IS determined by UVMR; (C) independent causal effects of remaining candidate mediators on IS after controlling for ALM by MVMR; (D) causal effects of potential mediators on ALM determined by UVMR. Different colors in the boxes indicate the magnitude of the *p*‐value. AF, atrial fibrillation; ALM, appendicular lean mass; CHD, coronary heart disease; CKD, chronic kidney disease; CRP, C‐reactive protein; DBP, diastolic blood pressure; FG, fasting glucose; FI, fasting insulin; HDL‐C, high‐density lipoprotein cholesterol; IS, ischemic stroke; IVW, inverse‐variance weighted; LDL‐C, low‐density lipoprotein cholesterol; ML, maximum likelihood; MR‐PRESSO^c^, outlier‐corrected MR‐PRESSO; MR‐PRESSO^r^, raw MR‐PRESSO; NAFLD, nonalcoholic fatty liver disease; MVMR, multivariable Mendelian randomization; SBP, systolic blood pressure; T2DM, type‐2 diabetes mellitus; TC, total cholesterol; TG, triglyceride; THG, two‐hour postprandial glucose; UVMR, univariable Mendelian randomization; WM, weighted median.

The flowchart illustrating our selection process is presented in Figure 3. Among the 21 potential mediators, each 1‐SD increase in ALM was significantly correlated with a reduced risk of developing hypertension (*β*: −0.095, 95% CI: −0.155 to −0.034, *p* = 2.34E‐03), T2DM (*β*: −0.118, 95% CI: −0.168 to −0.068, *p* = 5.95E‐06), AF (*β*: 0.284, 95% CI: 0.240 to 0.328, *p* = 3.45E‐36), CHD (*β*: −0.176, 95% CI: −0.230 to −0.121, *p* = 2.49E‐10), and NAFLD (*β*: −0.286, 95% CI: −0.492 to −0.081, *p* = 6.39E‐03), as well as lower levels of SBP (*β*: −0.863, 95% CI: −1.175 to −0.551, *p* = 6.02E‐08), DBP (*β*: −0.249, 95% CI: −0.424 to −0.074, *p* = 5.21E‐03), FI (*β*: −0.037, 95% CI: −0.050 to −0.023, *p* = 2.43E‐07), THG (*β*: −0.169, 95% CI: −0.212 to −0.126, *p* = 1.88E‐14), TC (*β*: −0.076, 95% CI: −0.108 to −0.044, *p* = 3.93E‐06), LDL‐C (*β*: −0.069, 95% CI: −0.103 to −0.036, *p* = 5.16E‐05), TG (*β*: −0.039, 95% CI: −0.069 to −0.008, *p* = 1.35E‐02), and CRP (*β*: −0.073, 95% CI: −0.091 to −0.056, *p* = 1.65E‐16) (Figure 4A; Table S9). However, due to significant horizontal pleiotropy between ALM and FI (p_intercept_ = 0.005), FI was excluded from further mediation analysis. No significant horizontal pleiotropy was observed between ALM and other mediators (all p_intercep_ ≥ 0.117) (Figure 4A; Table S10).

### 3.4 UVMR analyses for the causal impact of potential mediators on IS

Among the 12 potential mediators influenced by ALM, eight demonstrated a causal relationship with an elevated risk of IS. Our analyses provided robust evidence supporting the causal links between genetically determined increased risks of hypertension (*β*: 0.249, 95% CI: 0.174 to 0.325, *p* = 1.06E‐10), T2DM (*β*: 0.080, 95% CI: 0.051 to 0.109, *p* = 5.02E‐08), CHD (*β*: 0.166, 95% CI: 0.067 to 0.264, *p* = 1.02E‐03), and AF (*β*: 0.203, 95% CI: 0.162 to 0.245, *p* = 8.84E‐22), as well as elevated levels of SBP (*β*: 0.033, 95% CI: 0.029 to 0.039, *p* = 3.43E‐44), DBP (*β*: 0.046, 95% CI: 0.038 to 0.054, *p* = 6.01E‐31), TC (*β*: 0.111, 95% CI: 0.049 to 0.174, *p* = 4.56E‐04), and LDL‐C (*β*: 0.101, 95% CI: 0.037 to 0.164, *p* = 1.77E‐03), with IS (Figure 4B; Table S11). No evidence of horizontal pleiotropy was detected among these mediators and IS (all p_intercep_ ≥ 0.083) (Figure 4B; Table S12).

### 3.5 Calculation of the mediating effect and proportion of each mediator in the pathway between ALM and IS by two‐step MR analyses

**FIGURE 5:**
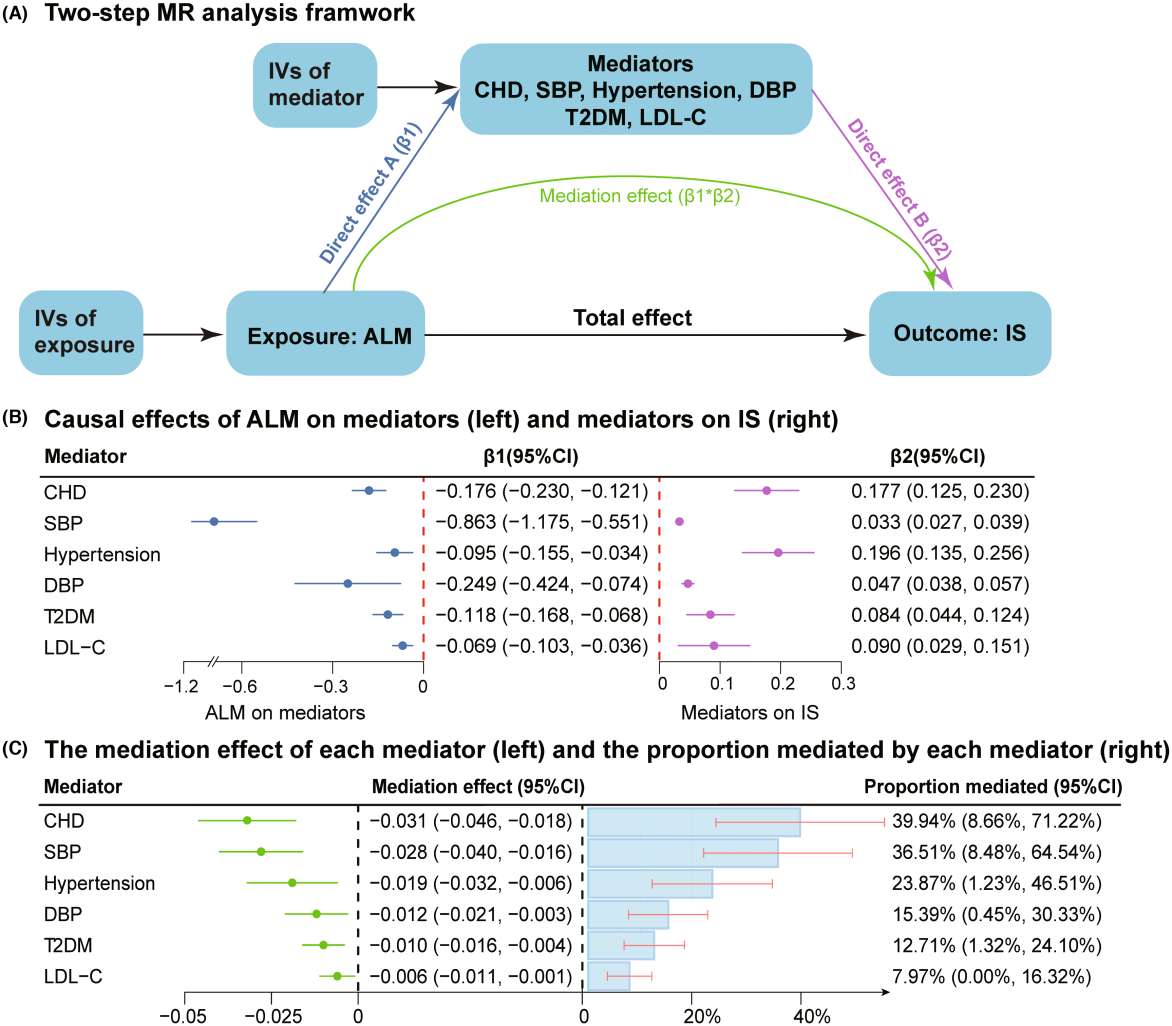
Two‐step MR estimates for the causal influence of ALM on IS via each mediator. (A) Two‐step MR analysis framework; (B) MR estimates for the causal effects of higher ALM on mediators (left) and the causal effects of mediators on IS with adjustment for ALM (right); (C) MR estimates for the mediating effect of each mediator (left) and the proportion mediated by each mediator (right). “Total effect (β0)” indicates the effect of higher ALM on IS, “direct effect A (β1)” indicates the effect of higher ALM on each mediator, “direct effect B (β2)” indicates the effect of each mediator on IS with adjustment for ALM, and “mediation effect” indicates the effect of higher ALM on IS via each mediator. Total effect (β0) and direct effect A (β1) were derived by the IVW method in UVMR (Table S9); direct effect B (β2) was from the IVW method in MVMR (Table S13). Standard errors for both mediating effects (β1*β2) and proportions (β1*β2/β0) were determined using the delta method. ALM, appendicular lean mass; CHD, coronary heart disease; CI, confidence interval; DBP, diastolic blood pressure; IS, ischemic stroke; IVs, instrumental variables; IVW, inverse variance weighted; LDL‐C, low‐density lipoprotein cholesterol; MR, Mendelian randomization; MVMR, multivariable Mendelian randomization; SBP, systolic blood pressure; T2DM, type‐2 diabetes mellitus; UVMR, univariable Mendelian randomization.

Subsequent MVMR analyses indicated that all eight aforementioned mediators exerted independent causal effects on IS, even after adjusting for ALM (Figure 4C; Table S13). Due to the directional inconsistency between mediating and total effects, AF was excluded from further analyses. Similarly, TC was removed owning to evidence of bidirectional causation with ALM (Figure 4D; Table S14). Ultimately, CHD, hypertension, SBP, DBP, T2DM, and LDL‐C were identified as significant mediators in the causal pathway from ALM to IS. More detailly, CHD mediated the largest proportion of the total effect (mediating proportion: 39.94%; 95% CI: 8.66%–71.22%), followed by SBP (36.51%; 95% CI: 8.48%–64.54%), hypertension (23.87%; 95% CI: 1.23%–46.51%), DBP (15.39%; 95% CI: 0.45%–30.33%), T2DM (12.71%; 95% CI: 1.32%–24.10%), and LDL‐C (7.97%; 95% CI: 0.00%–16.32%) (Figure 5).

## 4 DISCUSSION

In the present study, we employed MR analysis for the first time to evaluate the causal and independent impacts of sarcopenia‐related traits on the risk of IS. We identified key metabolic mediators, such as blood pressure regulation, glucose metabolism, and lipid metabolism, and quantified their mediating effects and proportions in the pathway from ALM to IS. Our comprehensive MR analyses, utilizing diverse methodologies, demonstrated that each 1‐SD increase in genetically proxied ALM was associated with a 7.5% reduction in the risk of IS. The protective effect persisted even after adjusting for BMI, WBFM, BF%, WHR, level of PA, and BMR. Notably, six of the 21 potential mediators, including CHD (mediating proportion: 39.94%), SBP (36.51%), hypertension (23.87%), DBP (15.39%), T2DM (12.71%), and LDL‐C (7.97%), were recognized to partially mediate the protective effect of ALM on IS. These findings offer new insights into the pathophysiological mechanisms by which low ALM contributes to CVD and highlight the importance of maintaining muscle mass to mitigate IS risk through metabolic syndrome mediation.

### 4.1 The interplay among sarcopenia, CVD, and stroke

Sarcopenia, exacerbated by global aging trends, is an increasingly critical health concern linked to CVD and CVD‐related mortality. Evidence from large prospective cohort studies, such as the UK Biobank and China's CHARLS, have consistently supported the association between sarcopenia and increased risk of CVD.^29^, ^30^ Furthermore, NHANES data suggest that individuals with sarcopenic obesity face the highest hazard ratios for all‐cause and CVD‐related mortality, with frailty and unhealthy metabolic status acting as crucial mediators.^31^ However, the specific relationship between sarcopenia and IS remains underexplored. Cross‐sectional and longitudinal studies based on China's CHARLS cohort have shown a higher stroke risk among sarcopenic individuals (OR = 2.70, HR = 1.67),^14^ yet this correlation has not been specifically investigated for the ischemic subtype. Additionally, research involving 12,237 Chinese participants demonstrated that sarcopenia defined by low HGS can predict the onset of stroke, both cross‐sectionally and longitudinally (OR = 1.600, HR = 1.893).^32^ Other studies focusing on populations with T2DM^12^ and hypertension^13^ also linked low muscle mass with an increased stroke risk. Nonetheless, the generalizability of these results is constrained by participant selection and the metabolic disorders present, compounded by often inadequate baseline data on PA and detailed body composition metrics, which are crucial for controlling confounding factors. Our study addressed these gaps by examining the causal relationships between three indicators of sarcopenia (ALM, HGS, and UWP) and the risk of IS. Unlike previous cohort studies, we observed no causal impact of HGS or UWP on IS. For instance, a study by Liu et al. associated declines in HGS with an increased incidence of stroke (OR = 1.600, HR = 1.893).^32^ By employing MVMR to adjust for four adipose‐related indices, level of PA, and BMR, we confirmed that higher ALM could independently reduce the risk of IS, irrespective of obesity‐related phenotypes and PA levels.

### 4.2 Potential pathophysiological mechanisms linking sarcopenia and metabolic disturbance

Research increasingly reveals that muscle loss is intricately linked to metabolic disturbances, with insulin resistance (IR), oxidative stress, and chronic inflammation posited as key mechanisms in this relationship.^33^ Skeletal muscles, the primary organs for glucose disposal, play essential roles in maintaining glucose homeostasis via insulin‐mediated signaling pathways. Normally, insulin binds to receptors on muscle cell membranes, initiating a cascade of phosphorylation reactions that lead to GLUT4 translocation, ultimately facilitating glucose uptake and glycogen synthesis.^34^ Loss of muscle mass diminishes insulin responsiveness, resulting in increased IR and disrupted glucose regulation. This is often compounded by compensatory hyperinsulinemia that accelerates the conversion of glucose into TGs, predominantly stored in the liver.^35^ Concurrently, muscle atrophy is associated with mitochondrial dysfunction and decreased BMR, promoting ectopic fat deposition and intracellular lipid accumulation.^36^ Sarcopenic obesity, characterized by reduced muscle mass coupled with abdominal fat, significantly worsens metabolic and cardiovascular complications.^37^ Lipid intermediates like diacylglycerol and ceramides, produced by intramuscular adipocytes, impair insulin signaling and intensify IR.^38^ In addition, both intramuscular and visceral adipocytes, as potent sources of pro‐inflammatory cytokines (e.g., TNF‐α, IL‐1β, IL‐6), contribute to a pro‐inflammatory milieu that exacerbates muscle fiber damage and oxidative stress.^39^ This systemic inflammation encourages the accumulation of M1 macrophages and myofibroblasts, which engage in ox‐LDL uptake, accelerating atherosclerosis progression.^33^, ^40^ Chronic inflammatory states further damage endothelial cells, activate angiotensin II, and promote hypertension.^41^ A meta‐analysis by Quan et al. indicated that elderly individuals with sarcopenia, especially those with sarcopenic obesity, are more susceptible to hypertension.^41^ Given these insights, our study treated metabolic disorders such as hypertension, T2DM, hyperlipidemia, and elevated CRP as potential mediators linking sarcopenia to IS. Consistent with previous studies, we found that genetically determined ALM influences blood pressure, lipid profiles, T2DM, systemic inflammation (CRP), and CHD. However, we failed to unveil the causal impact of systemic inflammation on IS. Although a statistically significant causal inference between ALM and FI (a key indicator of IR) was established, the presence of horizontal pleiotropy cast doubt on the reliability of this relationship.

### 4.3 Strategies to prevent muscle loss and sarcopenia

Our findings underscore the clinical and public health significance of preventing IS through maintaining or enhancing muscle mass, which is a practical approach for the general population. Effective non‐pharmacological interventions for preventing and potentially reversing sarcopenia include resistance training and adequate nutrition.^10^ Resistance training, widely recognized for its efficacy and affordability, increases muscle mass and strength, thereby enhancing physical performance and overall health.^42^, ^43^ This form of exercise is adaptable for various fitness levels, making it a versatile option for broad implementation. Nutritional strategies also play a critical role in sarcopenia prevention. Adequate dietary protein intake,^44^ vitamin D supplementation,^45^ consumption of polyunsaturated fatty acids,^46^ and adherence to an antioxidant‐rich diet^47^, ^48^ have all proven effective in mitigating muscle loss and delaying the progression of sarcopenia. These dietary recommendations can be seamlessly integrated into public health guidelines to improve muscle health across populations. Additionally, emerging evidence highlights the potential benefits of social support and environmental factors, such as reduced air pollution^49^ (specifically lower PM2.5 levels), in preventing sarcopenia and its related cognitive complications.^50^ These insights advocate for community‐based social support for the elderly and government‐level initiatives to improve air quality as essential strategies. Effective management of sarcopenia may substantially reduce the risk of IS, particularly in high‐risk groups.

### 4.4 Prospectives and outlooks

To translate our scientific findings into clinical practice and further reduce the incidence of IS, we propose several research directions. First, the generalizability of our conclusions needs to be validated across diverse prospective cohorts, encompassing various racial backgrounds, age groups, and genders. Second, the safety and efficacy of interventional measures such as resistance training and nutritional interventions require further exploration through prospective cohort studies and randomized controlled trials. These studies will determine whether interventions designed to prevent sarcopenia can indeed lower the risk of IS and other CVDs. Last, it is crucial to develop personalized strategies to identify which subgroups will benefit most from these interventions. We are optimistic that these efforts will significantly contribute to the prevention of IS.

### 4.5 Limitations

Indeed, there has been an ongoing debate pertaining to whether sarcopenia could increase the risk of IS. Studies often use diverse methodologies to measure muscle mass and employ various diagnostic criteria for sarcopenia, creating significant heterogeneity and comparability issues. Moreover, while observational studies aim to explore the causal association between sarcopenia and metabolism or health‐related outcomes, completely eliminating residual confounding is challenging. To address these issues, we utilized IVs from GWAS data sets and conducted UVMR and MVMR to investigate the causal impacts of sarcopenia on IS. However, there still exist some limitations in our analyses. We analyzed summary‐level GWAS data under the assumption of a linear relationship between variables, which may oversimplify the effects. Herein, exploration of potential nonlinear effects using individual‐level GWAS data could provide deeper insights. In addition, as sarcopenia predominantly affects the elderly, our MR analysis could not delineate this relationship within specific age groups but only in the general population. Nonetheless, through MR analysis, we could not elucidate this correlation within a specific age group but only in the whole population. Finally, using GWAS data from European populations may limit the applicability of our findings to non‐European groups due to differences in genetic backgrounds.

## 5 CONCLUSION

This MR study demonstrated a causal protective effect of higher ALM on IS, independent of PA and adiposity‐related indices. We also identified several cardiovascular factors including CHD, hypertension, T2DM, and hyperlipidemia as key mediators underlying the causal pathway between sarcopenia‐related traits and IS. Overall, our analyses underscore muscle mass as another modifiable factor for IS, finding that maintaining sufficient muscle mass could reduce the risk of such disease.

## AUTHOR CONTRIBUTIONS

Jiahao Song and Da Zhou wrote the first draft of the manuscript. Jiahao Song and Jingrun Li drew the required figures for this manuscript. Jiahao Song, Mengqi Wang, Lina Jia, and Duo Lan performed the material preparation, data collection, and statistical analysis. Da Zhou, Ran Meng, Xunming Ji, and Haiqing Song contributed to the manuscript revision. MR took full responsibility for the data, the analyses and interpretation, and the conduct of the research. All authors read and approved the final version.

## FUNDING INFORMATION

This work was supported by the National Natural Science Foundation of China (grant numbers: 82101390 and 82171297).

## CONFLICT OF INTEREST STATEMENT

It is noteworthy that this study only used publicly available summary‐level GWAS data and did not involve the utilization of individual‐level statistics, thus no ethical approval was required. No potential conflict of interest was reported by the authors.

## Supporting information


Data S1.

## Data Availability

The GWAS data sets analyzed during the current study are openly available from their corresponding original article based on their PMID.
