# Supplementary material for: The causal relationship between sarcopenia‐related traits and ischemic stroke: Insights from univariable and multivariable Mendelian randomization analyses
Source: CNS Neurosci Ther. 2024 May 16;30(5):e14759. doi: 10.1111/cns.14759 (PMC11099748; doi:10.1111/cns.14759)
Supplement: Supplementary file 1 — Data S1. [file CNS-30-e14759-s001.docx]

**Supplementary materials**

**Table of contents**

| **Table S1.** MR results for the causal effect of sarcopenia-related traits on ischemic stroke | ………………….2 |
| --- | --- |
| **Table S2.** Univariable MR pleiotropy and heterogeneity test for the association between sarcopenia-related traits and ischemic stroke | ……………….....3 |
| **Table S3.** MR Steiger test of directionality in the MR analysis for the effect of sarcopenia-related traits on ischemic stroke | ………………….4 |
| **Table S4.** MR results for the causal effect of sarcopenia-related traits on ischemic stroke after eliminating heterogeneity | ……………….…5 |
| **Table S5.** Univariable MR pleiotropy and heterogeneity test for the association between sarcopenia-related traits and ischemic stroke after eliminating heterogeneity | ………………….7 |
| **Table S6.** MR results for the causal effect of ischemic stroke on sarcopenia-related traits | ………….………7 |
| **Table S7.** Univariable MR pleiotropy and heterogeneity test for the association between ischemic stroke and sarcopenia-related traits | ……………….…8 |
| **Table S8.** Multivariable MR estimates for the independent effect of appendicular lean mass on ischemic stroke with adjustment for some confounders | ………………….9 |
| **Table S9.** Univariable MR estimates for the causal effects of appendicular lean mass on potential mediators | ………………….10 |
| **Table S10.** Univariable MR pleiotropy and heterogeneity test for the associations between ALM and potential mediators | ………………….13 |
| **Table S11.** Univariable MR estimates for the causal effects of remaining potential mediators on ischemic stroke | ………………….15 |
| **Table S12.** Univariable MR pleiotropy and heterogeneity test for the associations between remaining potential mediators and ischemic stroke | ……………....17 |
| **Table S13.** Multivariable MR estimates for the causal association between potential mediators and ischemic stroke with adjustment for appendicular lean mass | ……………....18 |
| **Table S14.** Univariable MR estimates for the causal effects of remaining potential mediators on appendicular lean mass | ……………....19 |
| **Table S15.** Univariable MR pleiotropy and heterogeneity test for the associations between remaining potential mediators and appendicular lean mass | ……………....21 |

**Table S1. MR results for the causal effect of sarcopenia-related traits on ischemic stroke**

| **Exposure** | **Method** | **nSNPs** | **F-statistics** | **OR (95%CI)** | **p-value** |
| --- | --- | --- | --- | --- | --- |
| Appendicular lean mass | Maximum likelihood | 616 | 87 | 0.925 (0.885-0.967) | <0.001 |
|  | IVW |  |  | 0.925 (0.879-0.974) | 0.003 |
|  | MR-Egger |  |  | 0.949 (0.840-1.073) | 0.406 |
|  | Weighted median |  |  | 0.961 (0.894-1.034) | 0.293 |
|  | MR-PRESSO^r^ |  |  | 0.925 (0.879-0.974) | 0.003 |
|  | MR-PRESSO^c^ (2 outliers) |  |  | 0.933 (0.886-0.981) | 0.006 |
| Left hand grip strength | Maximum likelihood | 153 | 24 | 0.924 (0.784-1.090) | 0.350 |
|  | IVW |  |  | 0.924 (0.785-1.088) | 0.344 |
|  | MR-Egger |  |  | 1.190 (0.619-2.286) | 0.603 |
|  | Weighted median |  |  | 0.948 (0.736-1.221) | 0.679 |
|  | MR-PRESSO^r^ |  |  | 0.924 (0.789-1.083) | 0.335 |
|  | MR-PRESSO^c^ (0 outliers) |  |  | 0.924 (0.789-1.083) | 0.335 |
| Right hand grip strength | Maximum likelihood | 165 | 24 | 0.997 (0.850-1.168) | 0.968 |
|  | IVW |  |  | 0.997 (0.841-1.181) | 0.970 |
|  | MR-Egger |  |  | 1.086 (0.552-2.137) | 0.811 |
|  | Weighted median |  |  | 1.058 (0.832-1.346) | 0.643 |
|  | MR-PRESSO^r^ |  |  | 0.997 (0.841-1.182) | 0.970 |
|  | MR-PRESSO^c^ (0 outliers) |  |  | 0.997 (0.841-1.182) | 0.970 |
| Usual walking pace | Maximum likelihood | 56 | 15 | 0.587 (0.418-0.823) | 0.002 |
|  | IVW |  |  | 0.600 (0.394-0.913) | 0.017 |
|  | MR-Egger |  |  | 0.525 (0.084-3.261) | 0.492 |
|  | Weighted median |  |  | 0.587 (0.323-0.901) | 0.018 |
|  | MR-PRESSO^r^ |  |  | 0.599 (0.394-0.912) | 0.021 |
|  | MR-PRESSO^c^ (0 outliers) |  |  | 0.599 (0.394-0.912) | 0.021 |

Abbreviations: MR=Mendelian randomization; SNP=single nucleotide polymorphism; OR=odds ratio; CI=confidence interval; IVW=inverse variance weighted; MR-PRESSO=Mendelian randomization pleiotropy residual sum and outlier.

**Table S2. Univariable MR pleiotropy and heterogeneity test for the association between sarcopenia-related traits and ischemic stroke**

| **Horizontal pleiotropy** | | | | | |
| --- | --- | --- | --- | --- | --- |
| **Exposure** | **Outcome** | **Method** | **Egger_intercept** | **Intercept_se** | **P_intercept_** |
| Appendicular lean mass | Ischemic stroke | MR-Egger | -6.24E-05 | 1.36E-03 | 0.674 |
| Left hand grip strength |  |  | -3.01E-03 | 3.85E-03 | 0.435 |
| Right hand grip strength |  |  | -1.03E-03 | 4.00E-03 | 0.797 |
| Usual walking pace |  |  | 1.26E-03 | 8.55E-03 | 0.883 |
| **Heterogeneity test** | | | | | |
| **Exposure** | **Outcome** | **Method** | **Q statistic** | **Q_df** | **P_heterogeneity_** |
| Appendicular lean mass | Ischemic stroke | IVW | 831 | 615 | 1.26E-08 |
|  |  | MR-Egger | 831 | 614 | 1.12E-08 |
| Left hand grip strength |  | IVW | 145 | 152 | 6.35E-01 |
|  |  | MR-Egger | 145 | 151 | 6.27E-01 |
| Right hand grip strength |  | IVW | 192 | 164 | 6.73E-02 |
|  |  | MR-Egger | 192 | 163 | 6.10E-02 |
| Usual walking pace |  | IVW | 88 | 55 | 3.17E-03 |
|  |  | MR-Egger | 88 | 54 | 2.43E-03 |

Abbreviations: MR=Mendelian randomization; IVW=inverse variance weighted.

**Table S3. MR Steiger test of directionality in the MR analysis for the effect of sarcopenia-related traits on ischemic stroke**

| **Exposure** | **Outcome** | **r^2^ exposure** | **r^2^ outcome** | **Correct causal direction** | **Steiger p-value** |
| --- | --- | --- | --- | --- | --- |
| Appendicular lean mass | Ischemic stroke | 0.134 | 1.95E-03 | TRUE | 0.00 |
| Left hand grip strength |  | 0.016 | 3.37E-04 | TRUE | 0.00 |
| Right hand grip strength |  | 0.017 | 4.71E-04 | TRUE | 0.00 |
| Usual walking pace |  | 0.006 | 2.36E-04 | TRUE | 1.25E-152 |

**Table S4. MR results for the causal effect of sarcopenia-related traits on ischemic stroke after eliminating heterogeneity.**

| **Exposure** | **Method** | **nSNPs** | **OR (95%CI)** | **p-value** |
| --- | --- | --- | --- | --- |
| Appendicular lean mass | Maximum likelihood | 562 | 0.935 (0.892-0.979) | 0.005 |
|  | IVW |  | 0.935 (0.893-0.979) | 0.005 |
|  | MR-Egger |  | 0.925 (0.828-1.033) | 0.168 |
|  | Weighted median |  | 0.954 (0.886-1.028) | 0.213 |
|  | MR-PRESSO^r^ |  | 0.935 (0.896-0.976) | 0.003 |
|  | MR-PRESSO^c^ (0 outliers) |  | 0.935 (0.896-0.976) | 0.003 |
| Left hand grip strength | Maximum likelihood | 149 | 0.867 (0.734-1.025) | 0.095 |
|  | IVW |  | 0.868 (0.736-1.025) | 0.095 |
|  | MR-Egger |  | 1.073 (0.556-2.071) | 0.834 |
|  | Weighted median |  | 0.883 (0.690-1.129) | 0.321 |
|  | MR-PRESSO^r^ |  | 0.868 (0.745-1.012) | 0.073 |
|  | MR-PRESSO^c^ (0 outliers) |  | 0.868 (0.745-1.012) | 0.073 |
| Right hand grip strength | Maximum likelihood | 156 | 0.980 (0.834-1.154) | 0.817 |
|  | IVW |  | 0.981 (0.835-1.152) | 0.816 |
|  | MR-Egger |  | 0.996 (0.528-1.878) | 0.991 |
|  | Weighted median |  | 1.041 (0.823-1.318) | 0.735 |
|  | MR-PRESSO^r^ |  | 0.981 (0.842-1.143) | 0.807 |
|  | MR-PRESSO^c^ (0 outliers) |  | 0.981 (0.842-1.143) | 0.807 |
| Usual walking pace | Maximum likelihood | 49 | 0.634 (0.444-0.906) | 0.012 |
|  | IVW |  | 0.640 (0.450-0.911) | 0.013 |
|  | MR-Egger |  | 0.525 (0.117-2.348) | 0.404 |
|  | Weighted median |  | 0.549 (0.326-0.926) | 0.024 |
|  | MR-PRESSO^r^ |  | 0.640 (0.450-0.911) | 0.017 |
|  | MR-PRESSO^c^ (0 outliers) |  | 0.640 (0.450-0.911) | 0.017 |

Abbreviations: MR=Mendelian randomization; SNP=single nucleotide polymorphism; OR=odds ratio; CI=confidence interval; IVW=inverse variance weighted; MR-PRESSO=Mendelian randomization pleiotropy residual sum and outlier.

**Table S5. Univariable MR pleiotropy and heterogeneity test for the association between sarcopenia-related traits and ischemic stroke after eliminating heterogeneity**

| **Horizontal pleiotropy** | | | | | |
| --- | --- | --- | --- | --- | --- |
| **Exposure** | **Outcome** | **Method** | **Egger_intercept** | **Intercept_se** | **P_intercept_** |
| Appendicular lean mass | Ischemic stroke | MR-Egger | 2.55E-04 | 1.23E-03 | 0.835 |
| Left hand grip strength |  |  | -2.51E-03 | 3.86E-03 | 0.517 |
| Right hand grip strength |  |  | -1.83E-04 | 3.75E-03 | 0.961 |
| Usual walking pace |  |  | 1.91E-03 | 7.14E-03 | 0.790 |
| **Heterogeneity test** | | | | | |
| **Exposure** | **Outcome** | **Method** | **Q statistic** | **Q_df** | **P_heterogeneity_** |
| Appendicular lean mass | Ischemic stroke | IVW | 500 | 561 | 0.969 |
|  |  | MR-Egger | 500 | 560 | 0.967 |
| Left hand grip strength |  | IVW | 127 | 148 | 0.896 |
|  |  | MR-Egger | 126 | 147 | 0.889 |
| Right hand grip strength |  | IVW | 140 | 155 | 0.798 |
|  |  | MR-Egger | 140 | 154 | 0.781 |
| Usual walking pace |  | IVW | 48 | 48 | 0.458 |
|  |  | MR-Egger | 48 | 47 | 0.420 |

Abbreviations: MR=Mendelian randomization; IVW=inverse variance weighted.

**Table S6. MR results for the causal effect of ischemic stroke on sarcopenia-related traits**

| **Outcome** | **Method** | **nSNPs** | **F-statistics** | **β (95%CI)** | **p-value** |
| --- | --- | --- | --- | --- | --- |
| Appendicular lean mass | Maximum likelihood | 12 | 811 | -0.009 (-0.029, 0.011) | 0.331 |
|  | IVW |  |  | -0.009 (-0.036, 0.018) | 0.515 |
|  | MR-Egger |  |  | -0.068 (-0.195, 0.059) | 0.320 |
|  | Weighted median |  |  | -0.014 (-0.041, 0.013) | 0.330 |
|  | MR-PRESSO^r^ |  |  | -0.009 (-0.036, 0.018) | 0.528 |
|  | MR-PRESSO^c^ (1 outliers) |  |  | -0.016 (-0.040, 0.007) | 0.205 |
| Left hand grip strength | Maximum likelihood | 14 | 800 | -0.012 (-0.026, 0.002) | 0.092 |
|  | IVW |  |  | -0.012 (-0.034, 0.010) | 0.287 |
|  | MR-Egger |  |  | -0.028 (-0.128, 0.072) | 0.601 |
|  | Weighted median |  |  | -0.004 (-0.028, 0.018) | 0.739 |
|  | MR-PRESSO^r^ |  |  | -0.012 (-0.034, 0.010) | 0.307 |
|  | MR-PRESSO^c^ (2 outliers) |  |  | 0.004 (-0.010, 0.018) | 0.610 |
| Right hand grip strength | Maximum likelihood | 14 | 800 | -0.007 (-0.021, 0.007) | 0.312 |
|  | IVW |  |  | -0.007 (-0.031, 0.017) | 0.541 |
|  | MR-Egger |  |  | -0.019 (-0.127, 0.089) | 0.735 |
|  | Weighted median |  |  | -0.007 (-0.027, 0.013) | 0.485 |
|  | MR-PRESSO^r^ |  |  | -0.007 (-0.031, 0.017) | 0.551 |
|  | MR-PRESSO^c^ (2 outliers) |  |  | 0.009 (-0.007, 0.024) | 0.261 |
| Usual walking pace | Maximum likelihood | 14 | 800 | -0.015 (-0.027, -0.003) | 0.014 |
|  | IVW |  |  | -0.015 (-0.031, 0.001) | 0.073 |
|  | MR-Egger |  |  | -0.052 (-0.125, 0.021) | 0.185 |
|  | Weighted median |  |  | -0.019 (-0.035, -0.001) | 0.037 |
|  | MR-PRESSO^r^ |  |  | -0.015 (-0.031, 0.001) | 0.097 |
|  | MR-PRESSO^c^ (0 outliers) |  |  | -0.015 (-0.031, 0.001) | 0.097 |

Abbreviations: MR=Mendelian randomization; SNP=single nucleotide polymorphism; CI=confidence interval; IVW=inverse variance weighted; MR-PRESSO=Mendelian randomization pleiotropy residual sum and outlier.

**Table S7. Univariable MR pleiotropy and heterogeneity test for the association between ischemic stroke and sarcopenia-related traits**

| **Horizontal pleiotropy** | | | | | |
| --- | --- | --- | --- | --- | --- |
| **Outcome** | **Exposure** | **Method** | **Egger_intercept** | **Intercept_se** | **P_intercept_** |
| Appendicular lean mass | Ischemic stroke | MR- Egger | 4.38E-03 | 4.71E-03 | 0.374 |
| Left hand grip strength |  |  | 1.15E-03 | 3.63E-03 | 0.757 |
| Right hand grip strength |  |  | 8.63E-04 | 3.91E-03 | 0.829 |
| Usual walking pace |  |  | 2.68E-03 | 2.59E-03 | 0.321 |
| **Heterogeneity test** | | | | | |
| **Outcome** | **Exposure** | **Method** | **Q statistic** | **Q_df** | **P_heterogeneity_** |
| Appendicular lean mass | Ischemic stroke | IVW | 24 | 11 | 1.32E-02 |
|  |  | MR-Egger | 22 | 10 | 1.14E-02 |
| Left hand grip strength |  | IVW | 35 | 13 | 9.53E-04 |
|  |  | MR-Egger | 34 | 12 | 5.88E-04 |
| Right hand grip strength |  | IVW | 40 | 13 | 1.44E-04 |
|  |  | MR-Egger | 40 | 12 | 7.99E-05 |
| Usual walking pace |  | IVW | 26 | 13 | 2.01E-02 |
|  |  | MR-Egger | 24 | 12 | 1.61E-02 |

Abbreviations: MR=Mendelian randomization; IVW=inverse variance weighted.

**Table S8. Multivariable MR estimates for the independent effect of appendicular lean mass on ischemic stroke with adjustment for some confounders**

| **Confounders** | **Outcome** | **Method** | **OR (95%CI)** | **p-value** | **nSNPs** | **Heterogeneity test** | | **Pleiotropy test** | | |
| --- | --- | --- | --- | --- | --- | --- | --- | --- | --- | --- |
|  |  |  |  |  |  | **Q-statistic** | **P-value** | **Egger-intercept** | **SE** | **p-value** |
| Body mass index | Ischemic stroke | MV-IVW | 0.904 (0.845, 0.970) | 4.87E-03 | 455 | 636 | 2.75E-05 | 1.11E-04 | 1.13E-03 | 0.921 |
|  |  | MVMR-Egger | 0.909 (0.807, 1.024) | 1.19E-01 |  | 636 | 2.31E-08 |  |  |  |
|  |  | MVMR-Median | 0.941 (0.859, 1.029) | 1.84E-01 |  | NA | NA |  |  |  |
|  |  | MVMR-Lasso | 0.942 (0.885, 1.002) | 5.61E-02 | 421 |  |  |  |  |  |
| Whole body fat mass | Ischemic stroke | MV-IVW | 0.916 (0.865, 0.969) | 2.45E-03 | 581 | 799 | 3.34E-09 | 1.53E-03 | 1.13E-03 | 0.175 |
|  |  | MVMR-Egger | 0.861 (0.774, 0.957) | 5.54E-03 |  | 796 | 4.05E-09 |  |  |  |
|  |  | MVMR-Median | 0.959 (0.889, 1.036) | 2.85E-01 |  | NA | NA |  |  |  |
|  |  | MVMR-Lasso | 0.930 (0.883, 0.978) | 5.24E-03 | 533 |  |  |  |  |  |
| Body fat percentage | Ischemic stroke | MV-IVW | 0.936 (0.884, 0.991) | 2.40E-02 | 606 | 895 | 1.09E-13 | 1.29E-03 | 1.21E-03 | 0.286 |
|  |  | MVMR-Egger | 0.889 (0.795, 0.993) | 3.80E-02 |  | 894 | 1.18E-13 |  |  |  |
|  |  | MVMR-Median | 0.971 (0.904, 1.042) | 4.21E-01 |  | NA | NA |  |  |  |
|  |  | MVMR-Lasso | 0.961 (0.915, 1.009) | 1.10E-01 | 560 |  |  |  |  |  |
| Waist-hip ratio | Ischemic stroke | MV-IVW | 0.940 (0.887,  0.994) | 2.99E-02 | 557 | 761 | 1.31E-08 | 9.29E-04 | 1.21E-03 | 0.444 |
|  |  | MVMR-Egger | 0.904 (0.807,  1.012) | 8.01E-02 | 557 | 760 | 1.24E-08 |  |  |  |
|  |  | MVMR-Median | 0.971 (0.901,  1.048) | 4.61E-01 | 557 | NA | NA |  |  |  |
|  |  | MVMR-Lasso | 0.960 (0.913,  1.010) | 1.13E-01 | 522 |  |  |  |  |  |
| Physical activity | Ischemic stroke | MV-IVW | 0.931 (0.882, 0.981) | 8.07E-03 | 565 | 779 | 3.82E-09 | -5.28E-04 | 1.45E-03 | 0.715 |
|  |  | MVMR-Egger | 0.951 (0.837, 1.081) | 4.43E-01 |  | 779 | 3.32E-09 |  |  |  |
|  |  | MVMR-Median | 0.979 (0.912, 1.051) | 5.64E-01 |  | NA | NA |  |  |  |
|  |  | MVMR-Lasso | 0.946 (0.903, 0.993) | 2.33E-02 | 530 |  |  |  |  |  |
| Basic metabolic rate | Ischemic stroke | MV-IVW | 0.820 (0.726, 0.927) | 1.43E-03 | 603 | 823 | 3.76E-09 | 1.21E-03 | 1.27E-03 | 0.341 |
|  |  | MVMR-Egger | 0.787 (0.679, 0.913) | 1.54E-03 |  | 822 | 3.80E-09 |  |  |  |
|  |  | MVMR-Median | 0.865 (0.739, 1.013) | 7.21E-02 |  | NA | NA |  |  |  |
|  |  | MVMR-Lasso | 0.835 (0.751, 0.930) | 9.81E-04 | 569 |  |  |  |  |  |

Abbreviations: MVMR= multivariable Mendelian randomization; OR=odds ratio; CI=confidence interval; SNP=single nucleotide polymorphism; SE=standard error; MV-IVW=multivariable inverse variance weighted model.

**Table S9. Univariable MR estimates for the causal effects of appendicular lean mass on potential mediators**

| **Potential mediators** | **Methods** | **nSNPs** | **β (95%CI)** | **p-value** |
| --- | --- | --- | --- | --- |
| Hypertension | IVW | 613 | -0.095 (-0.155, -0.034) | 2.34E-03 |
|  | Weighted median | 613 | -0.076 (-0.151, -0.001) | 4.73E-02 |
|  | Maximum likelihood | 613 | -0.096 (-0.138, -0.053) | 1.20E-05 |
|  | MR-Egger | 613 | -0.077 (-0.216, 0.063) | 2.81E-01 |
|  | MR-PRESSO^r^ | 613 | -0.095 (-0.156, -0.034) | 2.44E-03 |
|  | MR-PRESSO^c^ | 596 | -0.108 (-0.163, -0.053) | 1.36E-04 |
| Systolic blood pressure | IVW | 528 | -0.863 (-1.175, -0.551) | 6.02E-08 |
|  | Weighted median | 528 | -0.932 (-1.221, -0.643) | 2.51E-10 |
|  | Maximum likelihood | 528 | -0.862 (-1.007, -0.716) | 4.06E-31 |
|  | MR-Egger | 528 | -1.173 (-1.918, -0.427) | 2.15E-03 |
|  | MR-PRESSO^r^ | 528 | -0.863 (-1.175, -0.551) | 9.18E-08 |
|  | MR-PRESSO^c^ | 462 | -1.096 (-1.355, -0.837) | 1.43E-15 |
| Diastolic blood pressure | IVW | 525 | -0.249 (-0.424, -0.074) | 5.21E-03 |
|  | Weighted median | 525 | -0.240 (-0.419, -0.060) | 9.02E-03 |
|  | Maximum likelihood | 525 | -0.257 (-0.341, -0.173) | 2.11E-09 |
|  | MR-Egger | 525 | -0.541 (-0.962, -0.120) | 1.22E-02 |
|  | MR-PRESSO^r^ | 525 | -0.249 (-0.423, -0.075) | 5.40E-03 |
|  | MR-PRESSO^c^ | 450 | -0.242 (-0.387, -0.097) | 1.18E-03 |
| Type-2 Diabetes | IVW | 633 | -0.118 (-0.168, -0.068) | 5.95E-06 |
|  | Weighted median | 633 | -0.143 (-0.201, -0.085) | 1.26E-06 |
|  | Maximum likelihood | 633 | -0.119 (-0.149, -0.089) | 7.72E-15 |
|  | MR-Egger | 633 | -0.127 (-0.248, -0.006) | 3.98E-02 |
|  | MR-PRESSO^r^ | 633 | -0.118 (-0.169, -0.067) | 4.79E-06 |
|  | MR-PRESSO^c^ | 594 | -0.146 (-0.189, -0.103) | 1.53E-10 |
| Fasting insulin | IVW | 614 | -0.037 (-0.050, -0.023) | 2.43E-07 |
|  | Weighted median | 614 | -0.052 (-0.070, -0.033) | 2.25E-08 |
|  | Maximum likelihood | 614 | -0.036 (-0.046, -0.027) | 3.80E-15 |
|  | MR-Egger | 614 | -0.078 (-0.110, -0.046) | 2.12E-06 |
|  | MR-PRESSO^r^ | 614 | -0.037 (-0.051, -0.023) | 3.29E-07 |
|  | MR-PRESSO^c^ | 589 | -0.036 (-0.048, -0.024) | 1.67E-08 |
| HbA1c | IVW | 614 | -0.006 (-0.014, 0.003) | 2.00E-01 |
|  | Weighted median | 614 | -0.019 (-0.029, -0.008) | 5.89E-04 |
|  | Maximum likelihood | 614 | -0.006 (-0.012, 0.000) | 6.49E-02 |
|  | MR-Egger | 614 | -0.011 (-0.031, 0.009) | 2.71E-01 |
|  | MR-PRESSO^r^ | 614 | -0.006 (-0.014, 0.002) | 2.00E-01 |
|  | MR-PRESSO^c^ | 597 | -0.009 (-0.017, -0.001) | 3.11E-02 |
| Fasting glucose | IVW | 613 | -0.004 (-0.014, 0.008) | 5.50E-01 |
|  | Weighted median | 613 | -0.005 (-0.021, 0.010) | 4.91E-01 |
|  | Maximum likelihood | 613 | -0.003 (-0.011, 0.005) | 4.09E-01 |
|  | MR-Egger | 613 | -0.011 (-0.036, 0.015) | 4.18E-01 |
|  | MR-PRESSO^r^ | 613 | -0.003 (-0.015, 0.009) | 5.51E-01 |
|  | MR-PRESSO^c^ | 598 | -0.004 (-0.016, 0.008) | 4.98E-01 |
| Two-hour glucose | IVW | 615 | -0.169 (-0.212, -0.126) | 1.88E-14 |
|  | Weighted median | 615 | -0.174 (-0.239, -0.110) | 1.35E-07 |
|  | Maximum likelihood | 615 | -0.170 (-0.207, -0.132) | 1.15E-18 |
|  | MR-Egger | 615 | -0.235 (-0.334, -0.135) | 4.41E-06 |
|  | MR-PRESSO^r^ | 615 | -0.169 (-0.212, -0.126) | 7.34E-14 |
|  | MR-PRESSO^c^ | 611 | -0.179 (-0.220, -0.138) | 3.01E-16 |
| Total cholesterol | IVW | 386 | -0.076 (-0.108, -0.044) | 3.93E-06 |
|  | Weighted median | 386 | -0.086 (-0.133, -0.044) | 8.64E-05 |
|  | Maximum likelihood | 386 | -0.077 (-0.101, -0.052) | 7.76E-10 |
|  | MR-Egger | 386 | -0.020 (-0.096, 0.056) | 6.07E-01 |
|  | MR-PRESSO^r^ | 386 | -0.076 (-0.107, -0.045) | 5.36E-06 |
|  | MR-PRESSO^c^ | 380 | -0.073 (-0.104, -0.042) | 3.28E-06 |
| Low-density lipoprotein cholesterol | IVW | 388 | -0.069 (-0.103, -0.036) | 5.16E-05 |
|  | Weighted median | 388 | -0.066 (-0.111, -0.022) | 3.12E-03 |
|  | Maximum likelihood | 388 | -0.069 (-0.094, -0.044) | 5.54E-08 |
|  | MR-Egger | 388 | -0.036 (-0.115, 0.043) | 3.71E-01 |
|  | MR-PRESSO^r^ | 388 | -0.069 (-0.102, -0.036) | 6.24E-05 |
|  | MR-PRESSO^c^ | 381 | -0.056 (-0.087, -0.025) | 3.60E-04 |
| High density lipoprotein  cholesterol | IVW | 389 | -0.018 (-0.049, 0.014) | 2.76E-01 |
|  | Weighted median | 389 | -0.018 (-0.060, 0.023) | 3.80E-01 |
|  | Maximum likelihood | 389 | -0.018 (-0.041, 0.005) | 1.30E-01 |
|  | MR-Egger | 389 | -0.007 (-0.082, 0.069) | 8.62E-01 |
|  | MR-PRESSO^r^ | 389 | -0.018 (-0.049, 0.013) | 2.77E-01 |
|  | MR-PRESSO^c^ | 379 | -0.018 (-0.045, 0.009) | 2.10E-01 |
| Triglyceride | IVW | 389 | -0.039 (-0.069, -0.008) | 1.35E-02 |
|  | Weighted median | 389 | -0.057 (-0.102, -0.013) | 1.20E-02 |
|  | Maximum likelihood | 389 | -0.039 (-0.061, -0.016) | 7.55E-04 |
|  | MR-Egger | 389 | -0.013 (-0.086, 0.060) | 7.27E-01 |
|  | MR-PRESSO^r^ | 389 | -0.039 (-0.070, -0.008) | 1.39E-02 |
|  | MR-PRESSO^c^ | 380 | -0.040 (-0.067, -0.013) | 5.78E-03 |
| Atrial fibrillation | IVW | 636 | 0.284 (0.240, 0.328) | 3.45E-36 |
|  | Weighted median | 636 | 0.290 (0.234, 0.344) | 7.68E-25 |
|  | Maximum likelihood | 636 | 0.286 (0.256, 0.317) | 8.62E-77 |
|  | MR-Egger | 636 | 0.335 (0.229, 0.440) | 8.25E-10 |
|  | MR-PRESSO^r^ | 636 | 0.284 (0.239, 0.329) | 1.77E-32 |
|  | MR-PRESSO^c^ | 616 | 0.300 (0.259, 0.341) | 2.79E-41 |
| Coronary heart disease | IVW | 613 | -0.176 (-0.230, -0.121) | 2.49E-10 |
|  | Weighted median | 613 | -0.181 (-0.257, -0.106) | 2.23E-06 |
|  | Maximum likelihood | 613 | -0.177 (-0.218, -0.136) | 4.74E-17 |
|  | MR-Egger | 613 | -0.263 (-0.391, -0.136) | 5.88E-05 |
|  | MR-PRESSO^r^ | 613 | -0.176 (-0.231, -0.121) | 4.81E-10 |
|  | MR-PRESSO^c^ | 606 | -0.170 (-0.221, -0.119) | 2.20E-10 |
| Non-alcoholic fatty liver disease | IVW | 618 | -0.286 (-0.492, -0.081) | 6.39E-03 |
|  | Weighted median | 618 | -0.380 (-0.723, -0.037) | 2.97E-02 |
|  | Maximum likelihood | 618 | -0.280 (-0.486, -0.075) | 7.54E-03 |
|  | MR-Egger | 618 | -0.627 (-1.098, -0.155) | 9.41E-03 |
|  | MR-PRESSO^r^ | 618 | -0.286 (-0.492, -0.080) | 6.57E-03 |
|  | MR-PRESSO^c^ | 618 | -0.286 (-0.492, -0.080) | 6.57E-03 |
| Chronic kidney disease | IVW | 618 | -0.138 (-0.240, -0.035) | 8.49E-03 |
|  | Weighted median | 618 | -0.116 (-0.277, 0.045) | 1.58E-01 |
|  | Maximum likelihood | 618 | -0.136 (-0.238, -0.034) | 8.74E-03 |
|  | MR-Egger | 618 | 0.018 (-0.217, 0.253) | 8.80E-01 |
|  | MR-PRESSO^r^ | 618 | -0.138 (-0.240, -0.036) | 8.70E-03 |
|  | MR-PRESSO^c^ | 618 | -0.138 (-0.240, -0.036) | 8.70E-03 |
| Depression | IVW | 581 | -0.017 (-0.044, 0.010) | 2.08E-01 |
|  | Weighted median | 581 | 0.000 (-0.341, 0.341) | 1.000 |
|  | Maximum likelihood | 581 | -0.017 (-0.036, 0.002) | 7.61E-02 |
|  | MR-Egger | 581 | -0.020 (-0.083, 0.042) | 5.25E-01 |
|  | MR-PRESSO^r^ | 581 | -0.017 (-0.044, 0.010) | 2.09E-01 |
|  | MR-PRESSO^c^ | 568 | -0.013 (-0.037, 0.012) | 2.82E-01 |
| Anxiety | IVW | 618 | -0.015 (-0.068, 0.038) | 5.76E-01 |
|  | Weighted median | 618 | -0.013 (-0.094, 0.068) | 7.47E-01 |
|  | Maximum likelihood | 618 | -0.015 (-0.063, 0.033) | 5.46E-01 |
|  | MR-Egger | 618 | -0.028 (-0.150, 0.094) | 6.53E-01 |
|  | MR-PRESSO^r^ | 618 | -0.015 (-0.068, 0.038) | 5.76E-01 |
|  | MR-PRESSO^c^ | 618 | -0.015 (-0.068, 0.038) | 5.76E-01 |
| Cigarettes per day | IVW | 584 | 0.020 (-0.013, 0.052) | 2.34E-01 |
|  | Weighted median | 584 | 0.032 (-0.009, 0.072) | 1.26E-01 |
|  | Maximum likelihood | 584 | 0.020 (-0.004, 0.044) | 1.09E-01 |
|  | MR-Egger | 584 | 0.099 (0.024, 0.174) | 1.02E-02 |
|  | MR-PRESSO^r^ | 584 | 0.020 (-0.011, 0.051) | 2.35E-01 |
|  | MR-PRESSO^c^ | 579 | 0.022 (-0.009, 0.053) | 1.63E-01 |
| Alcoholic drinks per day | IVW | 579 | 0.009 (-0.003, 0.021) | 1.45E-01 |
|  | Weighted median | 579 | 0.004 (-0.011, 0.019) | 6.29E-01 |
|  | Maximum likelihood | 579 | 0.009 (0.001, 0.018) | 3.80E-02 |
|  | MR-Egger | 579 | 0.017 (-0.012, 0.046) | 2.58E-01 |
|  | MR-PRESSO^r^ | 579 | 0.009 (-0.003, 0.021) | 1.46E-01 |
|  | MR-PRESSO^c^ | 579 | 0.009 (-0.003, 0.021) | 1.46E-01 |
| C-reactive protein | IVW | 575 | -0.073 (-0.091, -0.056) | 1.65E-16 |
|  | Weighted median | 575 | -0.082 (-0.101, -0.064) | 2.71E-18 |
|  | Maximum likelihood | 575 | -0.073 (-0.082, -0.064) | 3.79E-53 |
|  | MR-Egger | 575 | -0.074 (-0.114, -0.033) | 4.26E-04 |
|  | MR-PRESSO^r^ | 575 | -0.073 (-0.091, -0.055) | 1.13E-15 |
|  | MR-PRESSO^c^ | 527 | -0.073 (-0.087, -0.059) | 8.83E-21 |

Abbreviations: MR=Mendelian randomization; SNP=single nucleotide polymorphism; CI=confidence interval; IVW=inverse variance weighted; MR-PRESSO=Mendelian randomization pleiotropy residual sum and outlier.

.

**Table S10. Univariable MR pleiotropy and heterogeneity test for the associations between ALM and potential mediators**

| **Horizontal pleiotropy test** | | | | |
| --- | --- | --- | --- | --- |
| **Exposure** | **Method** | **Egger_intercept** | **Intercept_se** | **p_intercept_** |
| Hypertension | MR Egger | -4.37E-04 | 1.57E-03 | 0.781 |
| Systolic blood pressure | MR Egger | 7.42E-03 | 8.26E-03 | 0.370 |
| Diastolic blood pressure | MR Egger | 6.93E-03 | 4.65E-03 | 0.137 |
| Type-2 diabetes | MR Egger | 2.18E-04 | 1.32E-03 | 0.869 |
| Fasting insulin | MR Egger | 1.02E-03 | 3.62E-04 | 0.005 |
| HbA1c | MR Egger | 1.38E-04 | 2.26E-04 | 0.542 |
| Fasting glucose | MR Egger | 1.79E-04 | 2.92E-04 | 0.540 |
| Two-hour glucose | MR Egger | 1.63E-03 | 1.13E-03 | 0.149 |
| Total cholesterol | MR Egger | -1.38E-03 | 8.61E-04 | 0.110 |
| Low-density lipoprotein cholesterol | MR Egger | -8.12E-04 | 8.96E-04 | 0.365 |
| High density lipoprotein | MR Egger | -2.72E-04 | 8.58E-04 | 0.751 |
| Triglyceride | MR Egger | -6.30E-04 | 8.21E-04 | 0.443 |
| Atrial fibrillation | MR Egger | -1.22E-03 | 1.18E-03 | 0.299 |
| Coronary heart disease | MR Egger | 1.93E-03 | 1.44E-03 | 0.181 |
| Non-alcoholic fatty liver disease | MR Egger | 8.35E-03 | 5.31E-03 | 0.117 |
| Chronic kidney disease | MR Egger | -3.82E-03 | 2.64E-03 | 0.149 |
| Depression | MR Egger | 7.79E-05 | 7.09E-04 | 0.913 |
| Anxiety | MR Egger | 3.15E-04 | 1.38E-03 | 0.819 |
| Cigarettes per day | MR Egger | -1.94E-03 | 8.51E-04 | 0.023 |
| Alcohol drinks per day | MR Egger | -1.86E-04 | 3.24E-04 | 0.566 |
| C-reactive protein | MR Egger | 3.21E-06 | 4.47E-04 | 0.994 |
| **Heterogeneity test** | | | | |
| **Exposure** | **Method** | **Q statistic** | **Q_df** | **P_heterogeneity_** |
| Hypertension | IVW | 1268 | 612 | 5.32E-48 |
|  | MR-Egger | 1267 | 611 | 3.85E-48 |
| Systolic blood pressure | IVW | 2560 | 527 | 1.69E-263 |
|  | MR-Egger | 2556 | 526 | 3.63E-263 |
| Diastolic blood pressure | IVW | 2386 | 524 | 9.69E-235 |
|  | MR-Egger | 2376 | 523 | 2.34E-233 |
| Type-2 diabetes | IVW | 1811 | 632 | 3.84E-114 |
|  | MR-Egger | 1811 | 631 | 2.32E-114 |
| Fasting insulin | IVW | 1467 | 613 | 8.57E-72 |
|  | MR-Egger | 1448 | 612 | 1.33E-69 |
| HbA1c | IVW | 1288 | 613 | 3.64E-50 |
|  | MR-Egger | 1287 | 612 | 3.08E-50 |
| Fasting glucose | IVW | 1196 | 612 | 4.24E-40 |
|  | MR-Egger | 1195 | 611 | 3.62E-40 |
| Two-hour glucose | IVW | 817 | 614 | 6.56E-08 |
|  | MR-Egger | 814 | 613 | 8.08E-08 |
| Total cholesterol | IVW | 687 | 385 | 2.37E-19 |
|  | MR-Egger | 683 | 384 | 4.88E-19 |
| Low-density lipoprotein cholesterol | IVW | 704 | 387 | 9.10E-21 |
|  | MR-Egger | 703 | 386 | 9.45E-21 |
| High density lipoprotein | IVW | 758 | 388 | 3.53E-26 |
|  | MR-Egger | 758 | 387 | 2.64E-26 |
| Triglyceride | IVW | 730 | 388 | 2.89E-23 |
|  | MR-Egger | 729 | 387 | 2.73E-23 |
| Atrial fibrillation | IVW | 1405 | 635 | 3.35E-60 |
|  | MR-Egger | 1403 | 634 | 4.33E-60 |
| Coronary heart disease | IVW | 1036 | 593 | 1.47E-26 |
|  | MR-Egger | 1033 | 592 | 2.18E-26 |
| Non-alcoholic fatty liver disease | IVW | 624 | 617 | 4.14E-01 |
|  | MR-Egger | 621 | 616 | 4.31E-01 |
| Chronic kidney disease | IVW | 634 | 617 | 3.11E-01 |
|  | MR-Egger | 632 | 616 | 3.22E-01 |
| Depression | IVW | 1179 | 580 | 4.16E-43 |
|  | MR-Egger | 1179 | 579 | 2.93E-43 |
| Anxiety | IVW | 760 | 617 | 6.66E-05 |
|  | MR-Egger | 760 | 616 | 6.00E-05 |
| Cigarettes per day | IVW | 1056 | 583 | 9.62E-30 |
|  | MR-Egger | 1046 | 582 | 5.91E-29 |
| Alcohol drinks per day | IVW | 1170 | 578 | 1.89E-42 |
|  | MR-Egger | 1170 | 577 | 1.57E-42 |
| C-reactive protein | IVW | 2097 | 574 | 6.00E-172 |
|  | MR-Egger | 2097 | 573 | 3.14E-172 |

Abbreviations: MR=Mendelian randomization; appendicular lean mass=ALM; IVW=inverse variance weighted.

**Table S11. Univariable MR estimates for the causal effects of remaining potential mediators on ischemic stroke**

| **Potential mediators** | **Methods** | **nSNPs** | **β (95%CI)** | **p-value** |
| --- | --- | --- | --- | --- |
| Hypertension | IVW | 35 | 0.249 (0.174, 0.325) | 1.06E-10 |
|  | Weighted median | 35 | 0.225 (0.136, 0.313) | 6.74E-07 |
|  | Maximum likelihood | 35 | 0.262 (0.206, 0.317) | 2.66E-20 |
|  | MR-Egger | 35 | 0.151 (-0.124, 0.426) | 2.91E-01 |
|  | MR-PRESSO^r^ | 35 | 0.249 (0.173, 0.325) | 2.20E-07 |
|  | MR-PRESSO^c^ | 35 | 0.249 (0.173, 0.325) | 2.20E-07 |
| Systolic blood pressure | IVW | 443 | 0.033 (0.029, 0.039) | 3.43E-44 |
|  | Weighted median | 443 | 0.036 (0.030, 0.042) | 4.27E-29 |
|  | Maximum likelihood | 443 | 0.033 (0.030, 0.037) | 5.73E-70 |
|  | MR-Egger | 443 | 0.042 (0.030, 0.054) | 1.36E-11 |
|  | MR-PRESSO^r^ | 443 | 0.033 (0.029, 0.037) | 6.97E-37 |
|  | MR-PRESSO^c^ | 436 | 0.034 (0.030, 0.038) | 1.43E-41 |
| Diastolic blood pressure | IVW | 443 | 0.046 (0.038, 0.054) | 6.01E-31 |
|  | Weighted median | 443 | 0.046 (0.036, 0.057) | 1.29E-17 |
|  | Maximum likelihood | 443 | 0.046 (0.040, 0.053) | 6.26E-48 |
|  | MR-Egger | 443 | 0.055 (0.035, 0.074) | 6.87E-08 |
|  | MR-PRESSO^r^ | 443 | 0.046 (0.038, 0.054) | 3.23E-27 |
|  | MR-PRESSO^c^ | 440 | 0.049 (0.041, 0.057) | 7.66E-32 |
| Type-2 Diabetes | IVW | 164 | 0.080 (0.051, 0.109) | 5.02E-08 |
|  | Weighted median | 164 | 0.093 (0.043, 0.145) | 3.23E-04 |
|  | Maximum likelihood | 164 | 0.081 (0.053, 0.109) | 1.92E-08 |
|  | MR-Egger | 164 | 0.083 (0.023, 0.142) | 7.02E-03 |
|  | MR-PRESSO^r^ | 164 | 0.080 (0.051, 0.109) | 1.83E-07 |
|  | MR-PRESSO^c^ | 164 | 0.080 (0.051, 0.109) | 1.83E-07 |
| Two-hour glucose | IVW | 13 | 0.134 (-0.003, 0.272) | 5.55E-02 |
|  | Weighted median | 13 | 0.093 (-0.047, 0.233) | 1.91E-01 |
|  | Maximum likelihood | 13 | 0.140 (0.047, 0.234) | 3.24E-03 |
|  | MR-Egger | 13 | 0.041 (-0.340, 0.422) | 8.38E-01 |
|  | MR-PRESSO^r^ | 13 | 0.134 (-0.003, 0.271) | 7.97E-02 |
|  | MR-PRESSO^c^ | 12 | 0.110 (-0.012, 0.232) | 1.03E-01 |
| Total cholesterol | IVW | 83 | 0.111 (0.049, 0.174) | 4.56E-04 |
|  | Weighted median | 83 | 0.126 (0.039, 0.212) | 4.42E-03 |
|  | Maximum likelihood | 83 | 0.112 (0.063, 0.161) | 8.15E-06 |
|  | MR-Egger | 83 | 0.185 (0.085, 0.285) | 2.34E-04 |
|  | MR-PRESSO^r^ | 83 | 0.111 (0.048, 0.173) | 7.41E-04 |
|  | MR-PRESSO^c^ | 83 | 0.111 (0.048, 0.173) | 7.41E-04 |
| Low-density lipoprotein cholesterol | IVW | 77 | 0.101 (0.037, 0.164) | 1.77E-03 |
|  | Weighted median | 77 | 0.082 (0.000, 0.164) | 4.94E-02 |
|  | Maximum likelihood | 77 | 0.101 (0.053, 0.148) | 3.43E-05 |
|  | MR-Egger | 77 | 0.142 (0.049, 0.236) | 3.92E-03 |
|  | MR-PRESSO^r^ | 77 | 0.101 (0.038, 0.164) | 2.51E-03 |
|  | MR-PRESSO^c^ | 77 | 0.101 (0.038, 0.164) | 2.51E-03 |
| Triglyceride | IVW | 55 | -0.007 (-0.077, 0.063) | 8.47E-01 |
|  | Weighted median | 55 | 0.001 (-0.097, 0.098) | 9.90E-01 |
|  | Maximum likelihood | 55 | -0.007 (-0.069, 0.055) | 8.28E-01 |
|  | MR-Egger | 55 | -0.084 (-0.193, 0.026) | 1.40E-01 |
|  | MR-PRESSO^r^ | 55 | -0.007 (-0.078, 0.064) | 8.47E-01 |
|  | MR-PRESSO^c^ | 55 | -0.007 (-0.078, 0.064) | 8.47E-01 |
| Coronary heart disease | IVW | 38 | 0.166 (0.067, 0.264) | 1.02E-03 |
|  | Weighted median | 38 | 0.143 (0.055, 0.231) | 1.49E-03 |
|  | Maximum likelihood | 38 | 0.178 (0.133, 0.223) | 1.22E-14 |
|  | MR-Egger | 38 | 0.081 (-0.154, 0.315) | 5.04E-01 |
|  | MR-PRESSO^r^ | 38 | 0.166 (0.068, 0.264) | 2.23E-03 |
|  | MR-PRESSO^c^ | 32 | 0.157 (0.090, 0.224) | 8.47E-05 |
| Atrial fibrillation | IVW | 110 | 0.203 (0.162, 0.245) | 8.84E-22 |
|  | Weighted median | 110 | 0.194 (0.140, 0.247) | 8.81E-13 |
|  | Maximum likelihood | 110 | 0.208 (0.175, 0.241) | 1.59E-35 |
|  | MR-Egger | 110 | 0.165 (0.064, 0.266) | 1.82E-03 |
|  | MR-PRESSO^r^ | 110 | 0.203 (0.162, 0.244) | 3.70E-16 |
|  | MR-PRESSO^c^ | 108 | 0.195 (0.156, 0.234) | 1.29E-16 |
| Non-alcoholic fatty liver disease* | IVW | 11 | -0.016 (-0.038, 0.006) | 1.47E-01 |
|  | Weighted median | 11 | -0.029 (-0.058, 0.000) | 5.20E-02 |
|  | Maximum likelihood | 11 | -0.016 (-0.038, 0.005) | 1.42E-01 |
|  | MR-Egger | 11 | -0.030 (-0.074, 0.015) | 2.22E-01 |
|  | MR-PRESSO^r^ | 11 | -0.016 (-0.036, 0.004) | 1.55E-01 |
|  | MR-PRESSO^c^ | 11 | -0.016 (-0.036, 0.004) | 1.55E-01 |
| C-reactive protein | IVW | 221 | 0.041 (-0.013, 0.095) | 1.35E-01 |
|  | Weighted median | 221 | 0.004 (-0.072, 0.082) | 9.01E-01 |
|  | Maximum likelihood | 221 | 0.041 (-0.005, 0.088) | 8.33E-02 |
|  | MR-Egger | 221 | 0.003 (-0.070, 0.077) | 9.28E-01 |
|  | MR-PRESSO^r^ | 221 | 0.041 (-0.014, 0.096) | 1.36E-01 |
|  | MR-PRESSO^c^ | 221 | 0.041 (-0.014, 0.096) | 1.36E-01 |

*: SNPs were selected at the threshold of p<5E-06.

Abbreviations: MR=Mendelian randomization; SNP=single nucleotide polymorphism; CI=confidence interval; IVW=inverse variance weighted; MR-PRESSO=Mendelian randomization pleiotropy residual sum and outlier.

**Table S12. Univariable MR pleiotropy and heterogeneity test for the associations between remaining potential mediators and ischemic stroke**

| **Horizontal pleiotropy test** | | | | |
| --- | --- | --- | --- | --- |
| **Exposure** | **Method** | **Egger_intercept** | **Intercept_se** | **p_intercept_** |
| Hypertension | MR-Egger | 8.41E-03 | 1.15E-02 | 0.470 |
| Systolic blood pressure | MR-Egger | -3.00E-03 | 1.85E-03 | 0.105 |
| Diastolic blood pressure | MR-Egger | -1.68E-03 | 1.79E-03 | 0.347 |
| Type-2 Diabetes | MR-Egger | -2.15E-4 | 2.17E-03 | 0.921 |
| Two-hour glucose | MR-Egger | 7.24E-03 | 1.39E-02 | 0.614 |
| Total cholesterol | MR-Egger | -5.12E-03 | 2.82E-03 | 0.073 |
| Low-density lipoprotein cholesterol | MR-Egger | -3.45E-03 | 2.93E-03 | 0.242 |
| Triglyceride | MR-Egger | 4.84E-03 | 2.84E-03 | 0.083 |
| Coronary heart disease | MR-Egger | 9.03E-03 | 1.15E-02 | 0.437 |
| Atrial fibrillation | MR-Egger | 2.96E-03 | 3.63E-03 | 0.417 |
| Non-alcoholic fatty liver disease* | MR-Egger | 7.77E-03 | 1.12E-02 | 0.506 |
| C-reactive protein | MR-Egger | 1.87E-03 | 1.27E-03 | 0.141 |
| **Heterogeneity test** | | | | |
| **Exposure** | **Method** | **Q statistic** | **Q_df** | **P_heterogeneity_** |
| Hypertension | IVW | 70 | 34 | 3.12E-04 |
|  | MR-Egger | 68 | 33 | 2.86E-04 |
| Systolic blood pressure | IVW | 730 | 442 | 1.59E-16 |
|  | MR-Egger | 726 | 441 | 2.95E-16 |
| Diastolic blood pressure | IVW | 711 | 442 | 6.54E-15 |
|  | MR-Egger | 710 | 441 | 6.76E-15 |
| Type-2 Diabetes | IVW | 172 | 163 | 3.01E-01 |
|  | MR-Egger | 172 | 162 | 2.83E-01 |
| Two-hour glucose | IVW | 28 | 12 | 6.53E-03 |
|  | MR-Egger | 27 | 11 | 4.84E-03 |
| Total cholesterol | IVW | 133 | 82 | 3.08E-04 |
|  | MR-Egger | 128 | 81 | 6.93E-04 |
| Low-density lipoprotein cholesterol | IVW | 135 | 76 | 3.45E-05 |
|  | MR-Egger | 133 | 75 | 4.50E-05 |
| Triglyceride | IVW | 68 | 54 | 9.01E-02 |
|  | MR-Egger | 65 | 53 | 1.32E-01 |
| Coronary heart disease | IVW | 196 | 37 | 1.61E-23 |
|  | MR-Egger | 193 | 36 | 2.67E-23 |
| Atrial fibrillation | IVW | 182 | 109 | 1.37E-05 |
|  | MR-Egger | 181 | 108 | 1.32E-05 |
| Non-alcoholic fatty liver disease* | IVW | 9 | 10 | 5.43E-01 |
|  | MR-Egger | 8 | 9 | 4.94E-01 |
| C-reactive protein | IVW | 293 | 220 | 7.05E-04 |
|  | MR-Egger | 290 | 219 | 8.93E-04 |

*: SNPs were selected at the threshold of p<5E-06.

Abbreviations: MR=Mendelian randomization; IVW=inverse variance weighted.

**Table S13. MVMR estimates for the causal association between potential mediators and ischemic stroke with adjustment for appendicular lean mass**

| **Potential Mediators** | **Method** | **β (95%CI)** | **p-value** | **nSNPs** | **Heterogeneity test** | | **Pleiotropy test** | | |
| --- | --- | --- | --- | --- | --- | --- | --- | --- | --- |
|  |  |  |  |  | **Q-statistic** | **P-value** | **Egger-intercept** | **SE** | **p-value** |
| Hypertension | MV-IVW | 0.196 (0.135, 0.256) | 2.60E-10 | 565 | 727 | 3.46E-06 | -2.44E-04 | 1.38E-03 | 0.860 |
|  | MVMR-Egger | 0.196 (0.135, 0.256) | 2.69E-10 |  | 729 | 3.04E-06 |  |  |  |
| Systolic blood pressure | MV-IVW | 0.033 (0.027, 0.039) | 3.25E-29 | 531 | 802 | 1.43E-13 | -2.23E-03 | 1.15E-03 | 0.053 |
|  | MVMR-Egger | 0.033 (0.027, 0.039) | 1.16E-29 |  | 797 | 3.09E-13 |  |  |  |
| Diastolic blood pressure | MV-IVW | 0.047 (0.038, 0.057) | 2.93E-21 | 537 | 808 | 1.86E-13 | 6.48E-04 | 1.16E-03 | 0.575 |
|  | MVMR-Egger | 0.047 (0.038, 0.057) | 3.27E-21 |  | 808 | 1.63E-13 |  |  |  |
| Type-2 Diabetes | MV-IVW | 0.084 (0.044, 0.124) | 3.53E-05 | 567 | 762 | 5.54E-08 | -2.28E-04 | 1.31E-03 | 0.862 |
|  | MVMR-Egger | 0.084 (0.044, 0.124) | 3.65E-05 |  | 762 | 4.77E-08 |  |  |  |
| Total cholesterol | MV-IVW | 0.112 (0.049, 0.175) | 4.71E-04 | 385 | 558 | 1.16E-08 | 1.39E-04 | 1.66E-03 | 0.933 |
|  | MVMR-Egger | 0.112 (0.049, 0.175) | 4.80E-04 |  | 558 | 9.53E-08 |  |  |  |
| Low-density lipoprotein cholesterol | MV-IVW | 0.090 (0.029, 0.151) | 4.01E-03 | 387 | 567 | 3.96E-09 | -8.96E-04 | 1.70E-03 | 0.598 |
|  | MVMR-Egger | 0.088 (0.026, 0.149) | 5.18E-03 |  | 567 | 3.47E-09 |  |  |  |
| Coronary heart disease | MV-IVW | 0.177 (0.125, 0.230) | 4.09E-11 | 575 | 764 | 1.42E-07 | -8.45E-04 | 1.36E-03 | 0.534 |
|  | MVMR-Egger | 0.178 (0.126, 0.231) | 3.54E-11 |  | 763 | 1.31E-07 |  |  |  |
| Atrial fibrillation | MV-IVW | 0.216 (0.169, 0.263) | 2.13E-19 | 571 | 750 | 4.82E-07 | -1.45E-03 | 1.31E-03 | 0.267 |
|  | MVMR-Egger | 0.215 (0.168, 0.262) | 3.33E-19 |  | 748 | 5.12E-7 |  |  |  |

Abbreviations: MVMR=multivariable Mendelian randomization; CI=confidence interval; SNP=single nucleotide polymorphism; SE=standard error; MV-IVW=multivariable inverse variance weighted model.

**Table S14. Univariable MR estimates for the causal effects of remaining potential mediators on appendicular lean mass**

| **Potential mediators**  **(Exposures)** | **Methods** | **nSNPs** | **β (95%CI)** | **p-value** |
| --- | --- | --- | --- | --- |
| Hypertension | IVW | 26 | 0.010 (-0.021, 0.041) | 5.30E-01 |
|  | Weighted median | 26 | 0.022 (-0.002, 0.047) | 7.80E-02 |
|  | Maximum likelihood | 26 | 0.011 (-0.002, 0.023) | 9.60E-02 |
|  | MR-Egger | 26 | 0.061 (-0.063, 0.185) | 3.43E-01 |
|  | MR-PRESSO^r^ | 26 | 0.010 (-0.021, 0.041) | 5.36E-01 |
|  | MR-PRESSO^c^ | 18 | 0.019 (-0.003, 0.041) | 1.18E-01 |
| Systolic blood pressure | IVW | 378 | -0.001 (-0.003, 0.001) | 2.49E-01 |
|  | Weighted median | 378 | 0.001 (-0.001, 0.003) | 2.31E-01 |
|  | Maximum likelihood | 378 | -0.001 (-0.002, 0.000) | 5.11E-03 |
|  | MR-Egger | 378 | 0.006 (0.001, 0.010) | 1.18E-02 |
|  | MR-PRESSO^r^ | 378 | -0.001 (-0.003, 0.001) | 2.50E-01 |
|  | MR-PRESSO^c^ | 307 | -0.001 (-0.003, 0.001) | 1.05E-01 |
| Diastolic blood pressure | IVW | 374 | 0.000 (-0.003, 0.003) | 9.26E-01 |
|  | Weighted median | 374 | 0.003 (0.000, 0.006) | 4.77E-02 |
|  | Maximum likelihood | 374 | 0.000 (-0.001, 0.001) | 8.17E-01 |
|  | MR-Egger | 374 | 0.007 (-0.001, 0.015) | 9.68E-02 |
|  | MR-PRESSO^r^ | 374 | 0.000 (-0.004, 0.004) | 9.26E-01 |
|  | MR-PRESSO^c^ | 295 | 0.001 (-0.001, 0.003) | 5.78E-01 |
| Type-2 diabetes mellitus | IVW | 141 | -0.004 (-0.017, 0.010) | 5.71E-01 |
|  | Weighted median | 141 | 0.006 (-0.005, 0.017) | 3.06E-01 |
|  | Maximum likelihood | 141 | -0.004 (-0.01, 0.002) | 1.67E-01 |
|  | MR-Egger | 141 | -0.015 (-0.042, 0.012) | 2.69E-01 |
|  | MR-PRESSO^r^ | 141 | -0.004 (-0.018, 0.010) | 5.72E-01 |
|  | MR-PRESSO^c^ | 114 | -0.008 (-0.018, 0.002) | 1.62E-01 |
| Total cholesterol | IVW | 71 | -0.024 (-0.045, 0.002) | 3.25E-02 |
|  | Weighted median | 71 | -0.001 (-0.018, 0.016) | 8.97E-01 |
|  | Maximum likelihood | 71 | -0.024 (-0.034, -0.014) | 7.94E-07 |
|  | MR-Egger | 71 | -0.001 (-0.034, 0.033) | 9.70E-01 |
|  | MR-PRESSO^r^ | 71 | -0.024 (-0.046, -0.002) | 3.60E-02 |
|  | MR-PRESSO^c^ | 61 | -0.026 (-0.048, -0.004) | 1.68E-02 |
| Low-density lipoprotein cholesterol | IVW | 67 | -0.020 (-0.069, 0.002) | 6.90E-02 |
|  | Weighted median | 67 | -0.010 (-0.026, 0.007) | 2.53E-01 |
|  | Maximum likelihood | 67 | -0.020 (-0.029, -0.011) | 1.23E-05 |
|  | MR-Egger | 67 | 0.002 (-0.028, 0.032) | 9.09E-01 |
|  | MR-PRESSO^r^ | 67 | -0.020 (-0.042, 0.002) | 7.35E-02 |
|  | MR-PRESSO^c^ | 56 | -0.025 (-0.045, -0.005) | 8.40E-03 |
| Coronary heart disease | IVW | 30 | 0.003 (-0.015, 0.021) | 7.68E-01 |
|  | Weighted median | 30 | 0.000 (-0.015, 0.015) | 9.71E-01 |
|  | Maximum likelihood | 30 | 0.003 (-0.006, 0.012) | 5.27E-01 |
|  | MR-Egger | 30 | -0.005 (-0.045, 0.035) | 8.17E-01 |
|  | MR-PRESSO^r^ | 30 | 0.003 (-0.015, 0.021) | 7.70E-01 |
|  | MR-PRESSO^c^ | 26 | 0.003 (-0.011, 0.017) | 6.58E-01 |
| Atrial fibrillation | IVW | 98 | 0.007 (-0.006, 0.019) | 3.18E-01 |
|  | Weighted median | 98 | 0.002 (-0.009, 0.013) | 7.41E-01 |
|  | Maximum likelihood | 98 | 0.007 (0.001, 0.013) | 2.47E-02 |
|  | MR-Egger | 98 | 0.001 (-0.024, 0.025) | 9.61E-01 |
|  | MR-PRESSO^r^ | 98 | 0.007 (-0.007, 0.021) | 3.21E-01 |
|  | MR-PRESSO^c^ | 82 | 0.008 (-0.001, 0.018) | 9.60E-02 |

Abbreviations: MR=Mendelian randomization; SNP=single nucleotide polymorphism; CI=confidence interval; IVW=inverse variance weighted; MR-PRESSO=Mendelian randomization pleiotropy residual sum and outlier.

**Table S15. Univariable MR pleiotropy and heterogeneity test for the associations between remaining potential mediators and appendicular lean mass**

| **Horizontal pleiotropy test** | | | | |
| --- | --- | --- | --- | --- |
| **Exposure** | **Method** | **Egger_intercept** | **Intercept_se** | **p_intercept_** |
| Hypertension | MR-Egger | -4.50E-03 | 5.38E-03 | 0.412 |
| Systolic blood pressure | MR-Egger | -2.28E-03 | 6.98E-04 | 0.001 |
| Diastolic blood pressure | MR-Egger | -1.29E-03 | 7.26E-04 | 0.076 |
| Type-2 Diabetes | MR-Egger | 9.37E-04 | 1.02E-03 | 0.342 |
| Total cholesterol | MR-Egger | -1.72E-03 | 9.89E-04 | 0.086 |
| Low-density lipoprotein cholesterol | MR-Egger | -1.99E-03 | 1.02E-03 | 0.054 |
| Coronary heart disease | MR-Egger | 8.37E-04 | 2.04E-03 | 0.686 |
| Atrial fibrillation | MR-Egger | 5.54E-04 | 1.01E-03 | 0.585 |
| **Heterogeneity test** | | | | |
| **Exposure** | **Method** | **Q statistic** | **Q_df** | **P_heterogeneity_** |
| Hypertension | IVW | 182 | 25 | 7.35E-26 |
|  | MR-Egger | 177 | 24 | 2.53E-25 |
| Systolic blood pressure | IVW | 2264 | 377 | 7.04E-266 |
|  | MR-Egger | 2201 | 376 | 5.63E-255 |
| Diastolic blood pressure | IVW | 2435 | 373 | 9.61E-299 |
|  | MR-Egger | 2414 | 372 | 2.22E-295 |
| Type-2 Diabetes | IVW | 865 | 140 | 7.01E-105 |
|  | MR-Egger | 860 | 139 | 2.30E-104 |
| Total cholesterol | IVW | 378 | 70 | 8.45E-44 |
|  | MR-Egger | 362 | 69 | 2.44E-41 |
| Low-density lipoprotein cholesterol | IVW | 393 | 66 | 4.64E-48 |
|  | MR-Egger | 371 | 65 | 1.80E-44 |
| Coronary heart disease | IVW | 126 | 29 | 4.49E-14 |
|  | MR-Egger | 125 | 28 | 2.80E-14 |
| Atrial fibrillation | IVW | 494 | 97 | 1.88E-54 |
|  | MR-Egger | 492 | 96 | 1.54E-54 |

Abbreviations: MR=Mendelian randomization; IVW=inverse variance weighted.
